# Evaluation of heat stress effects on cellular and transcriptional adaptation of bovine granulosa cells

**DOI:** 10.1186/s40104-019-0408-8

**Published:** 2020-02-18

**Authors:** Adnan Khan, Jinhuan Dou, Yachun Wang, Xiaolong Jiang, Muhammad Zahoor Khan, Hanpeng Luo, Tahir Usman, Huabin Zhu

**Affiliations:** 1 Key Laboratory of Animal Genetics, Breeding, and Reproduction, MARA; National Engineering Laboratory for Animal Breeding, College of Animal Science and Technology, China Agricultural University, Beijing, 100193 People’s Republic of China; 2 Embryo Biotechnology and Reproduction Laboratory, Institute of Animal Sciences, Chinese Academy of Agricultural Sciences, Beijing, China; 3 College of Veterinary Sciences and Animal Husbandry, Abdul Wali Khan University, Mardan, 23200 Pakistan

**Keywords:** Bovine granulosa cells, Differentially expressed genes, Follicles, Heat stress, RNA-Seq

## Abstract

**Background:**

Heat stress is known to affect follicular dynamics, oocyte maturation, and fertilization by impairing steroidogenic ability and viability of bovine granulosa cell (bGCs). The present study explored the physiological and molecular response of bGCs to different heat stress intensities *in-vitro*. We exposed the primary bGCs to heat stress (HS) at 39 °C, 40 °C and 41 °C along with control samples (38 °C) for 2 h. To evaluate the impact of heat stress on bGCs, several *in vitro* cellular parameters including cell apoptosis, intracellular reactive oxygen species (ROS) accumulation and *HSP70* kinetics were assessed by flow cytometry, florescence microscopy and western blot, respectively. Furthermore, the ELISA was performed to confirm the 17β-estradiol (E_2_) and progesterone (P_4_) levels. In addition, the RNA sequencing (RNA-Seq) method was used to get the molecular based response of bGCs to different heat treatments.

**Results:**

Our findings revealed that the HS significantly decreased the cell viability, E_2_ and P_4_ levels in bGCs, whereas, increased the cellular apoptosis and ROS. Moreover, the RNA-Seq experiments showed that all the treatments (39 °C, 40 °C and 41 °C) significantly regulated many differentially expressed genes (DEGs) i.e. *BCL2L1, STAR, CYP11A1, CASP3, SOD2, HSPA13*, and *MAPK8IP1* and pathways associated with heat stress, apoptosis, steroidogenesis, and oxidative stress. Conclusively, our data demonstrated that the impact of 40 °C treatment was comparatively detrimental for cell viability, apoptosis and ROS accumulation. Notably, a similar trend of gene expression was reported by RT-qPCR for RNA-seq data.

**Conclusions:**

Our study presented a worthy strategy for the first time to characterize the cellular and transcriptomic adaptation of bGCs to heat stress (39, 40 and 41 °C) *in-vitro*. The results infer that these genes and pathways reported in present study could be useful candidates/indicators for heat stress research in dairy cattle. Moreover, the established model of bGCs to heat stress in the current study provides an appropriate platform to understand the mechanism of how heat-stressed bGCs can affect the quality of oocytes and developing embryo.

## Background

Mammalian ovarian follicle, consisting of an oocyte that undergoes series of biological events including ovulation, fertilization, and formation of an embryo is surrounded by granulosa and theca cells producing signals and hormones to enable the oocyte to develop [1]. During follicle development, granulosa cells (GCs) replicate, secrete hormones, and provide a critical microenvironment for follicular growth [2]. Proliferation and differentiation of GCs is essential for normal follicular growth, development of oocyte, ovulation, and luteinization [3, 4].

Heat stress is one of the environmental factors that have harmful effects on the function of ovaries [5] and subsequently decreases the developmental ability of oocytes to be fertilized and further develop the competent embryo [6]. It significantly reduced the production of estradiol, and stenedione synthesis by theca cells [7], inhibited proliferation and induced apoptosis in swine granulosa cells [8]. In support of this, heat stress during *in vitro* fertilization increased polyspermy and decreased fertilization success by disrupting the antipolyspermy system in oocytes [9], suggesting that heat stress during fertilization mainly affects the oocyte and its developmental competence. Mammalian cells are known to respond to a wide range of environmental stressors in a variety of ways including; heat shock response protein expression [10], unfolded protein response (UPR) [11] and oxidative stress response [12] to support cell survival under suboptimal conditions. Cells may use constitutive induced heat shock proteins (HSPs), molecular chaperones in response to heat stress that facilitate the synthesis, folding, assembly, and transportation of stress-denatured proteins [13]. Heat shock 70 kDa protein (HSP70) is a major stress protein induced in mouse GCs by high temperature [9]. Increasing evidence suggests that heat stress induces intracellular ROS concentration [14], resulting in apoptosis of granulosa cells in the mouse [15]. In addition, ROS may subsequently alter the development of bovine embryos during *in-vitro* oocyte maturation [16].

RNA sequencing (RNA-Seq) has emerged as an innovative method for both mapping and quantifying transcriptome signatures associated with traits [17]. One of the most biologically relevant applications of RNA-Seq is the comparison of mRNA transcriptome across samples from diseased vs. normal individuals, or other specific experimental conditions [18]. The usage of high-throughput RNA sequencing technology has become a powerful tool and a standard method for the measurement and comparison of gene expression levels in a myriad of species and conditions [19]. Therefore, in our study, we employed RNA-Seq to characterize the complete transcriptome of bGC and facilitate the discovery of differentially expressed genes as well as novel genes and pathways under heat stress.

**Fig. 1:**
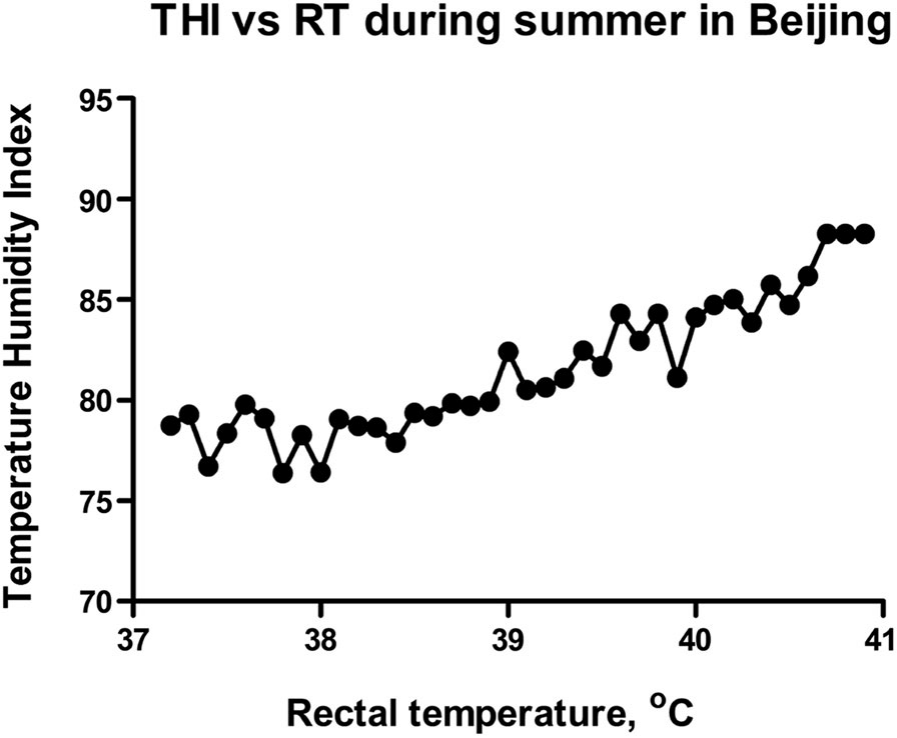
Temperature humidity index can affect body rectal temperature: Evaluation of change in rectal body temperature (RT) with increase in percent temperature humidity index (%THI)

This study was conducted in Beijing, China. Temperature levels were selected for the experiment to treat the granulosa cells, isolated from the ovaries of cattle that were well adapted to the local environment. For instance, we attempted to select experimental temperature levels that were relevant to the physiological body temperatures of cattle under HS in Beijing. During the summer, we collected the data from many dairy farms in Beijing, showing how environmental temperature-humidity index (THI) can affect rectal body temperature (RT). We found that in summer, body temperature may rise to 41 °C (Fig. 1). Therefore, we evaluated the effects of the four temperature levels [38 (control), 39, 40, and 41 °C] on the physiological traits and transcriptomic gene expression profile in bGCs.

Moreover, while much is now known about the effects of various factors on normal granulosa cells [14, 20], to the best of our understanding, no attempt has been taken so far to propose a molecular mechanism or explore gene interactions and molecular pathways related to heat stress response in bGCs at different heat intensities. We hypothesize that, relative to control, bGCs exposed to heat stress will experience alterations both in physiological traits and expression of key genes and pathways required for normal cellular functions. Therefore, the present study was aimed to explore cellular adaptation, generate global gene expression profile of bovine granulosa cells under normal and heat-stressed state and identify molecular pathways significantly regulated in heat-stressed bGCs.

## Methods

### Collection of bovine ovaries and granulosa cells isolation

Ovaries from dairy cattle were collected from a local abattoir and transported to the laboratory in thermally insulated bottles containing sterile physiological saline with 100 U/mL Penicillin and 0.1 mg/mL Streptomycin, at 28–30 °C within 2 h of harvesting. After washing with warm 0.9% NaCl solution three times and rinsing in 70% warm ethanol for 30 s, ovaries were washed thrice with warm Dulbecco’s Phosphate-Buffered Saline (DPBS). For isolation of BGCs, small healthy follicles (with a diameter of 2–6 mm) were selected using an 18-gauge sterile needle (B-Braun, Germany) and transferred into 15 mL conical centrifuge tubes (Corning, NY, USA). The follicular fluid containing cumulus-oocyte complexes (COCs) and granulosa cells was filtered using a filter with a diameter of 70 μm leaving COCs on the filter. The filtrate with granulosa cells was carefully transferred into 15 mL conical centrifuge tubes, centrifuged at 1500×*g* for 5 min. The supernatant of the follicular fluid was discarded by aspiration, and granulosa cells were washed thrice in phosphate-buffered saline (PBS), pH 7.4. The GCs were then resuspended in DMEM/F-12 (Gibco, Life Technologies Inc., Grand Island, NY, USA) supplemented with 1% penicillin-streptomycin and 10% fetal bovine serum (FBS, Gibco, Life Technologies Inc., Grand Island, NY, USA).

### Granulosa cell culture and heat treatment

Granulosa cells (6 × 10^6^ cells per well) were precultured in a 6-well plate (Starlab, Hamburg, Germany) with 2 mL of DMEM/F-12 (Gibco, Life Technologies Inc., Grand Island, NY, USA) culturing media supplemented with 1% penicillin-streptomycin and 10% FBS (Gibco, Life Technologies Inc., Grand Island, NY, USA) at 38 °C (optimal and physiologically relevant temperature for culturing mammalian ovarian cells) under 5% CO_2_ in humidified air.

After 48 h of preculture, cells were attached to the bottom of the wells with the confluence of more than 80%; the medium was replaced with the fresh medium of the same composition. GCs were then cultured at temperatures control group (38 °C), or heat treatment groups (39, 40 and 41 °C) for 2 h, and then the cells were cultured at 38 °C for 12 h. The cells and culture media were collected for further analysis immediately after the culture. Following heat treatments, cultured GCs were harvested using 0.25% trypsin-EDTA (Sigma-Aldrich Chemie, Taufkirchen, Germany).

### Western blot analysis of *HSP70*

Western blot analysis was used in all samples to determine the expression of inducible *HSP70* under heat stress. Granulosa cells from each group were washed thrice with 0.1% PVA/PBS, lysed in RIPA lysis buffer (Beyotime, Shanghai, China) containing protease inhibitors. Total protein concentration was measured with Protein Assay (Bio-Rad, 500–0002) and a spectrophotometer at 595 nm (Beckman, DU 530). Proteins were denatured at 100 °C for 10 min, separated by SDS-PAGE (12% acrylamide gel containing 0.1% SDS) and transferred onto a nitrocellulose membrane (BioTraceNT, Pall Corp., Port Washington, NY, USA). Membranes were then blocked with 5% (w/v) skim milk in Tris-buffered saline (TBS) containing 0.1% Tween 20 (TBST) at 37 °C for 1 h. The membranes were incubated overnight at 4 °C with primary antibodies against *HSP70* and *β-actin* after three washes in TBST. All primary antibodies were purchased from Cell Signaling Technology (Beverly, MA, USA), and diluted to a concentration of 1:1000. After washing in TBST thrice, the membranes were incubated at room temperature for 1 h with horseradish peroxidase (HRP)-conjugated secondary antibody (Zhongshan Biotechnology, Beijing, China). Based on the manufacturer’s instructions, the protein bands were detected using enhanced chemiluminescence (ECL) detection kit (Tanon, Shanghai, China) and analyzed by densitometry using Image J 1.44p software. The final data exported from Image J was analyzed in Microsoft Excel. Western blot in triplicate was performed for all samples.

### Determination of estradiol and progesterone by ELISA

All of the culture media was collected from controlled and heat treated groups and then estimated the levels of P_4_ and E_2__._ The concentrations of P_4_ and E_2_ were determined using an estrogen and progesterone enzyme-linked immunosorbent (ELISA) kits (ENZO life sciences, Germany) according to the manufacturer’s instruction.

### Determination of intracellular ROS production

About 2 × 10^4^ granulosa cells were cultured in 96-well plates. After growing to a confluence of more than 80%, GCs were incubated at 38, 39, 40 and 41 °C for 2 h. Following incubation, cells were stained with 10 μmol/L H2DCFDA fluorescent probe (6-carboxy- 2′,7′-dichlorodihydrofluorescein diacetate) (Invitrogen, Carlsbad, CA, USA) for 30 min at 38 °C in the dark. GCs samples were then washed once in 0.1% PVA/DPBS, and the images were captured immediately under a fluorescence microscope (Olympus, Tokyo, Japan) equipped with a CoolSNAP HQ CCD camera (Photometrics/Roper Scientific, Inc., Tucson, AZ, USA). Image J 1.44p software was used to analyze the fluorescence intensity.

### Estimation of granulosa cells apoptosis

Bovine GCs were harvested by enzymatic digestion using trypsin and washed three times with preheated PBS. Using the FITC-Annexin V/dead cell apoptosis kit (Life Technologies Inc., Grand Island, NY, USA), APC annexin V/PI double staining was performed to evaluate the granulosa cell apoptosis according to the manufacturer’s instructions before being analyzed by flow cytometry. The data were analyzed by Flowjo software (version Win64–10.4.0).

### Estimation of cell viability

Cultured and heat treated GCs were trypsinized, collected, and washed with warm PBS. The GCs were then passing through APC annexin V/PI double staining by utilizing the FITC-Annexin V/dead cell apoptosis kit (Life Technologies Inc., Grand Island, NY, USA) to assess the cell viability and apoptosis. Samples were washed with 1× annexin-binding buffer for 5 min in accordance with the manufacturer’s instructions and incubated in 490 μL 1× annexin-binding buffer supplemented with 10 μL annexin V conjugate at room temperature in the dark for 15 min. A laser-scanning confocal microscope (TCS SP8, Leica, Germany) was used to determine the number of early apoptotic and dead cells.

### RNA extraction for RNA-Seq

The RNA was isolated from bovine granulosa cells using RNA kit (Tiangen, Beijing, China) according to the manufacturer’s instructions. RNA samples were treated with RNase free DNase I to avoid DNA contamination. RNA degradation and contamination were detected by 1% agarose gels. The RNA concentration was assessed using NanoPhotometer spectrophotometer (Implen, CA, USA). The extracted RNA was stored at − 80 °C and all 12 samples (three from each group) were sent to the company (Gene Denovo Biotechnology Co. Guangzhou, China) for RNA-Seq analysis.

### Library construction for RNA-Seq

Three samples from each group were selected for library preparation. For the RNA sample preparations, A total amount of 2 μg RNA per sample was used as input material. Using NEBNext® Ultra™ RNA Library Prep Kit for Illumina® (#E7530L, NEB, USA),sequencing libraries were generated following the manufacturer’s recommendations and index codes were added to assign sequences to each sample. Briefly, using Oligo (dT) magnetic beads mRNA was purified from total RNA. Fragmentation was done in NEBNext First-Strand Synthesis Reaction Buffer (5×) using divalent cations at high temperature. First strand cDNA was synthesized using random hexamer primer, and RNase H. DNA polymerase I, RNase H, dNTP, and buffer were used to synthesize second-strand cDNA. Then the fragments cDNA were purified with QiaQuick PCR extraction kit, end repaired, poly(A) added, and ligated to Illumina sequencing adapters. The ligation products were size selected by agarose gel electrophoresis, PCR amplified and sequenced by Gene Denovo Biotechnology Co. (Guangzhou, China) using Illumina HiSeq**™** 2500 and generated 150 bp paired-end reads.

### Bioinformatics and statistical analysis of RNA-Seq

Raw reads generated by Illumina Hiseq™2500 were initially processed to get clean reads through the following three steps. i) Removing reads with adaptors contamination; ii) Discarding reads containing more than 10% of unknown nucleotides (N); iii) Removing low quality reads containing more than 50% of low quality (Q-value≤20) bases using Next Generation Sequencing (NGS) Quality Control Toolkit version 2.3.3. The filtered reads of each sample was individually mapped to 48370 reference mRNAs from the *Bos taurus* reference genome (UMD3.1) obtained from Ensembl (ftp: //ftp. Ensembl. org/pub/release-73/fasta/bos_taurus/dna/) HISAT2 software version 2.0.1 (http://ccb.jhu.edu/software/hisat2). The transcripts were then assembled and quantified using StringTie software version 1.2.2 (http://ccb.jhu.edu/software/stringtie). Using StringTie, transcript files generated were added to a single-merged transcriptome annotation to merge transcripts from different replicas of a group into a comprehensive set of transcripts, and then merge the transcripts from multiple groups into a finally a comprehensive set of transcripts for further downstream differential expression analysis. Differentially expressed genes (DEGs) and transcripts were identified among different sample groups using Ballgown. Ballgown was used as a pipeline package in the R programming language version 3.2.2 (https://www.r-project.org) and the Bioconductor software was used for plotting raw data, normalization and downstream statistical modeling. Gene expression values were calculated by counting the number of fragments per kilobase of transcript per million mapped fragments (FPKM), and Cuffdiff was applied to measure significant differences among the four groups. The result was sorted in Microsoft Excel. DEGs were subjected to Gene Ontology (GO) enrichment and Kyoto Encyclopedia of genes and genomes (KEGG) pathway analyses using the Molecule Annotation System http://david.abcc.ncifcrf.gov/) [21]. Using STRING software (version 10), a network was constructed with the genes involved in the significant pathways in order to generate a protein-protein interaction (PPI) network and to predict physical/functional PPIs. The heat map was constructed using ggplot two packages in R (version 3.2).

### Quantitative reverse transcription PCR (RT-qPCR) validation for RNA-Seq analysis

RT-qPCR was conducted to confirm the results of RNA-Seq. Total RNA was extracted from three biological replicates of control and heat-treated granulosa cells as described above and was reverse transcribed using first strand cDNA synthesis Kit (Thermo Fisher Scientific, Germany) with oligo (dT) 18 primers according to the manufacturer protocols. The expression levels were checked for 15 genes. Primer3 web version 4.0.0 (http://bioinfo.ut.ee/primer3/) and Primer blast (http://www.ncbi.nlm.nih.gov/tools/primer-blast/) were used for designing gene-specific primers and are shown in (Additional file 5). The RT-qPCR was conducted using iTaq™ Universal SYBR® Green Supermix (Bio-Rad Laboratories GmbH, Germany) in Applied Biosystem® StepOnePlus™ (Applied biosystems, CA, USA). A reaction volume of 20 μL with 7.4 μL of ddH_2_O, 0.3 μL of forwarding primer, 0.3 μL of reverse primer, 10 μL of 1× SYBR Green master mix (Bio-Rad Laboratories GmbH, Germany), and 2 μL of cDNA template was used. The Light Cycler 480 instrument (Roche, Germany) was used for performing qPCR. The second derivative maximum method was employed for data acquiring and subjected for further analysis. Using GAPDH as a reference gene, the 2^−ΔΔCT^ method was used to calculate gene expression levels, [21].

### Statistical analysis

Data are expressed as mean values ± SEM. Statistical analysis was carried out using SPSS 16.0. The difference between control and heat-treated groups for cell apoptosis, cell viability, steroidogenesis, ROS accumulation as well as the RT-qPCR results were analyzed using One-way ANOVA followed by multiple comparisons post hoc test. Differences were considered to be statistically significant at *P* < 0.05.

## Results

### Heat stress induces *HSP70* expression in bovine granulosa cells

**Fig. 2:**
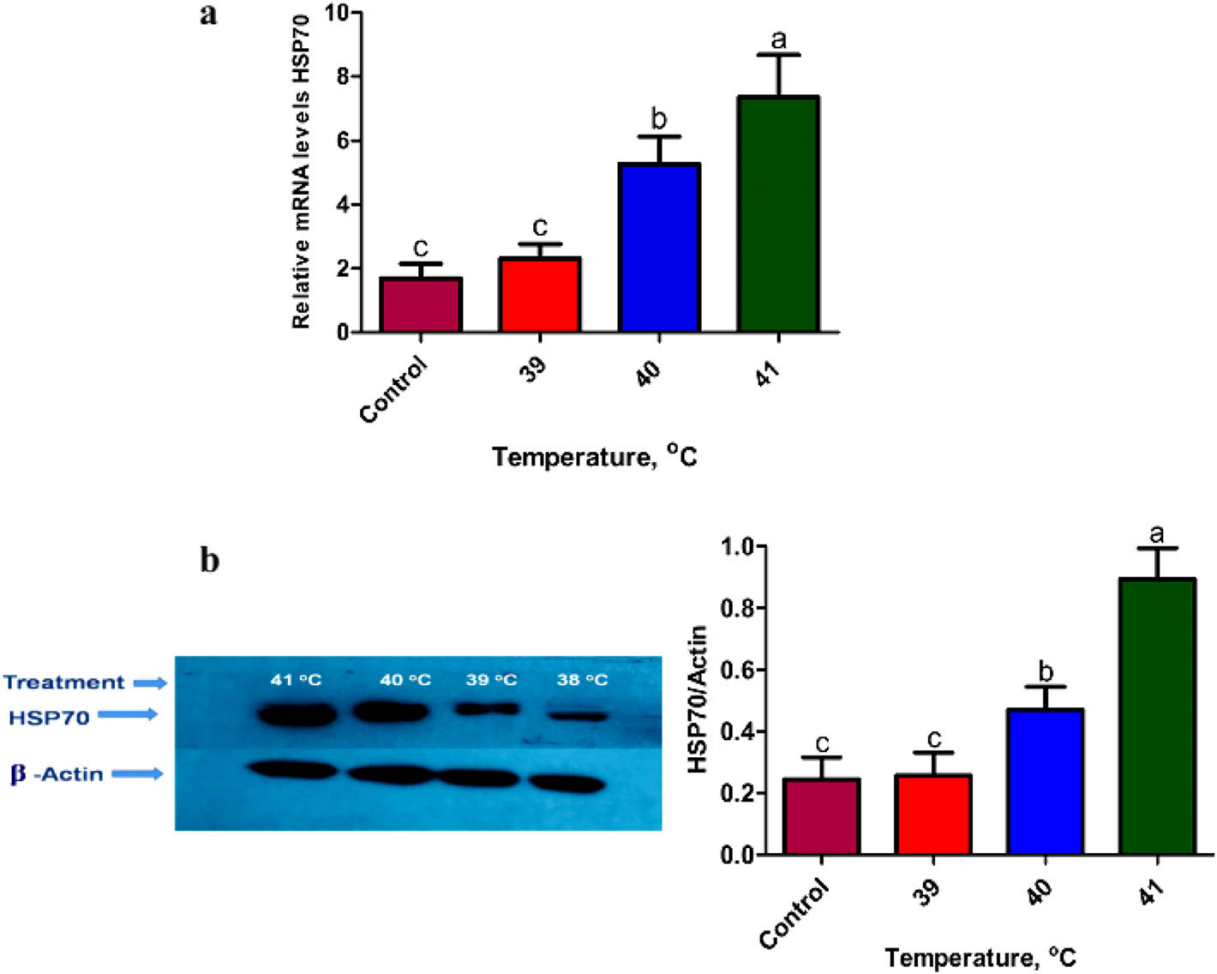
Heat stress induces *HSP70* expression in bovine granulosa cells: mRNA as well as protein expression of *HSP70* (**a**, **b**) in bovine granulosa cells cultured under heat stress (39, 40 and 41 °C) and corresponding control (38 °C). β-ACTIN was used to normalize the expression of target protein expression of *HSP70*. The results are expressed as the mean ± SEM of *n* = 3. Superscripts (a, b, c) show significant difference, *P* < 0.05

Bovine granulosa cells were heat treated at different temperature levels (Control, 39, 40 and 41 °C) for 2 h duration to investigate the effect of heat stress on the expression level of *HSP70* in bGCs. We performed western blot and RT-qPCR to check relative abundance of *HSP70* both on mRNA and protein level. Our results showed that the expression of *HSP70* between control and heat stressed group (39 °C) was not significantly different. However, the expression of *HSP70* was significantly up-regulated in bGCs under heat stress at 40 °C and 41 °C post-treatment (Fig. 2a, b).

### Heat stress exposure elevates bovine granulosa cell apoptosis

**Fig. 3:**
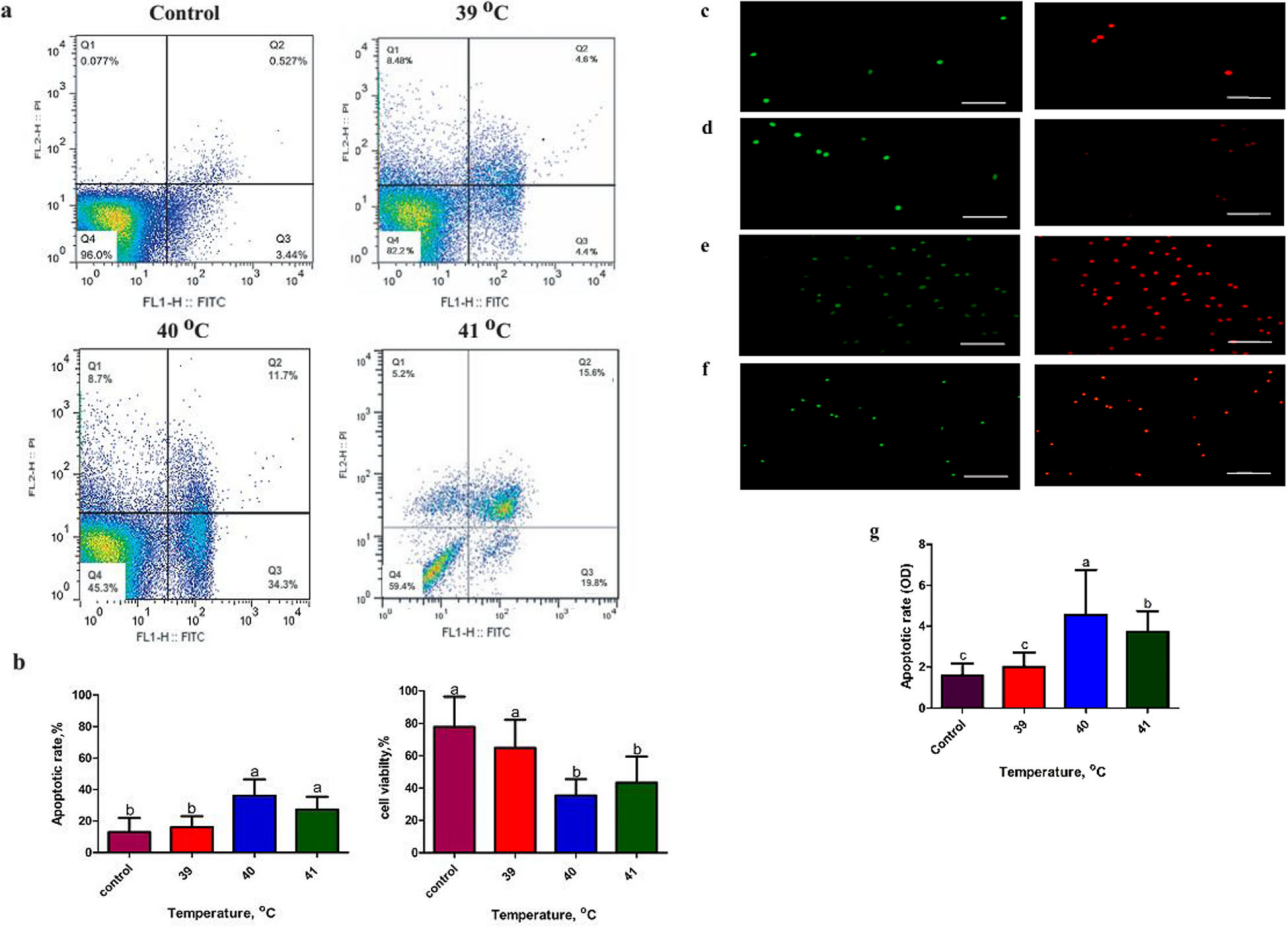
Heat stress exposure elevates bovine granulosa cell apoptosis and decreases viability: Flow cytometric analysis of bovine granulosa cells (bGCs) cultured under heat stress (39, 40 and 41 °C) and corresponding control (38 °C) (**a**, **b**). The analyzed cell counts for apoptosis and viability are indicated on the *Y*-axis and the temperature treatments are indicated on the *X*-axis. Data shown as means ± SEM, *n* = 3, *P* < 0.05. Fluorescent photomicrographs of bovine granulosa cells (bGCs) stained with 2′,7′-dichlorofluorescin diacetate (H_2_DCFDA) were shown control (38 °C) (**c**) and heat stress (39, 40 and 41 °C) (**d**, **e**, **f**, respectively). The images shown are representative of the three independent image acquisitions. **g** Quantitative analysis of relative fluorescence emission. Values are expressed as mean ± SEM of *n* = 3. Superscripts (a, b, c) show significant difference, *P* < 0.05

The bGCs apoptotic rate was estimated by flow cytometry (FCM) and fluorescence microscopy. It was found that the apoptotic rate (early + late apoptosis) of the GCs was significantly higher (*P* < 0.05) in the heat-treated groups (Fig. 3a, b). During cell culture, bGCs were exposed to heat stress for 2 h with a range of temperatures (Control, 39, 40 and 41 °C). Following heat stress exposure, the cell apoptotic rate was increased in temperature-dependent fashion. As shown in Fig. 3a, b, the apoptotic rate (46%) of bGCs was significantly (*P* < 0.05) higher at 40 °C than other treatments. However, with respect to 40 °C, the apoptotic rate of GCs was lower at 41 °C (35.4%). Heat treatment of 39 °C did not change apoptotic rate (9%) significantly than control group(3.96%). Similar effect of heat stress was noticed for cell viability. Significantly (*P* < 0.05) lower cell viability was found at 40 °C (45.3%) as compared to control (96%) and 39 °C (82.2%). No significant difference were noticed between 40 °C (45.3%) and 41 °C (59.4.3%) as shown in Fig. 3a, b. Fluorescence microscopy was also conducted to estimate the apoptotic rate and viability of GCs and found that the relative fluorescence emissions were higher when GCs were exposed to 40 °C than the control group. However, the 39 °C treatment group did not show any significant (*P* < 0.05) difference with the control group. Likewise, fluorescence microscopy showed that after 40 °C, the apoptotic rate decreased significantly (*P* < 0.05) in the heat stress group at 41 °C (Fig. 3c, d, e, f, g).

### Effects of heat stress on E_2_ and P_4_ secretion by bovine granulosa cells

**Fig. 4:**
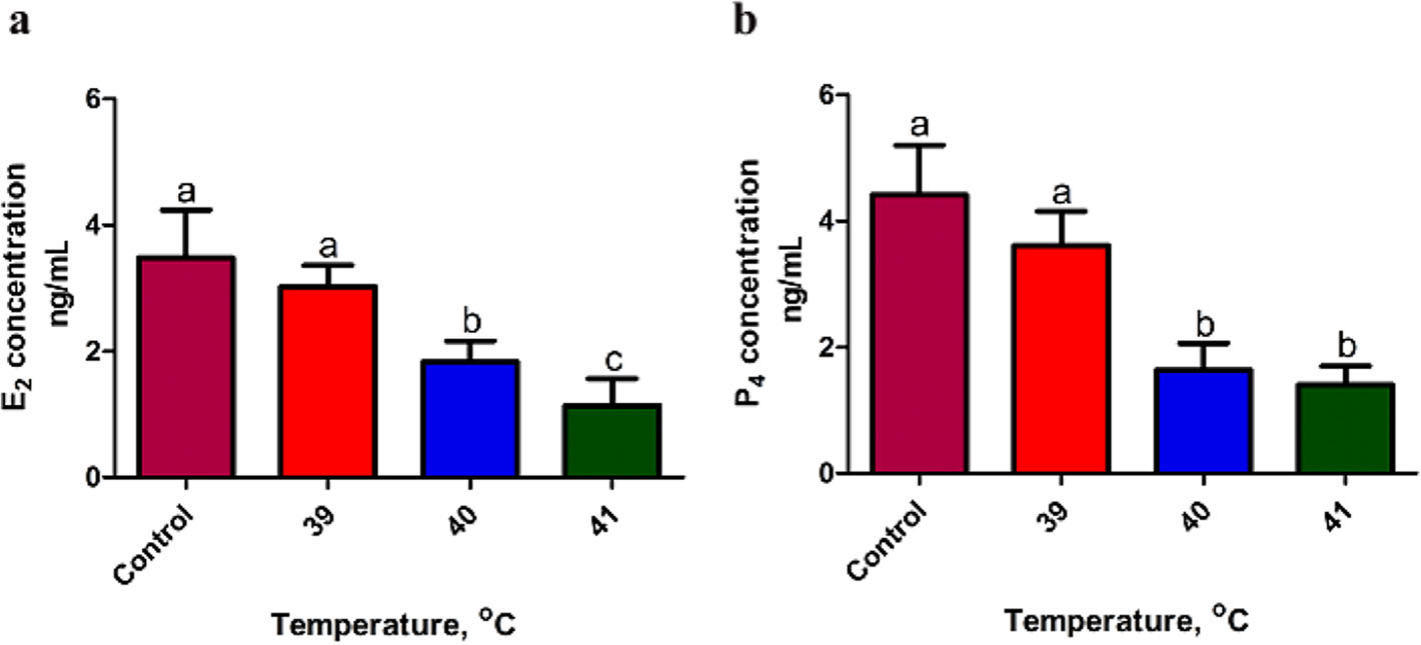
Effects of heat stress on E_2_ and P_4_ secretion by bovine granulosa cells: Concentration of E_2_ (**a**) and P_4_ (**b**) in culture media of bovine granulosa cells (bGCs) cultured under heat stress (39, 40 and 41 °C) and corresponding control (38 °C). Values are expressed as mean ± SEM of *n* = 3. Superscripts (a, b, c) show significant difference, *P* < 0.05

The concentration of E_2_ (Fig. 4a) in the heat-treated groups (40 and 41 °C) was significantly lower (*P* < 0.05) than the control and 39 °C group in the culture media. However, heat treated group at 39 °C did not show a significant difference with the control group. Furthermore, a significant (*P* < 0.05) difference was noticed between treatment groups of 40 and 41 °C. Similar secretion pattern was also observed for P_4_ with the significant difference (*P* < 0.05) between control and heat-treated groups (40 and 41 °C) (Fig. 4b). However, no significant difference was noticed between 40 and 41 °C treated groups.

### Heat stress enhanced intracellular ROS accumulation in bovine granulosa cells

**Fig. 5:**
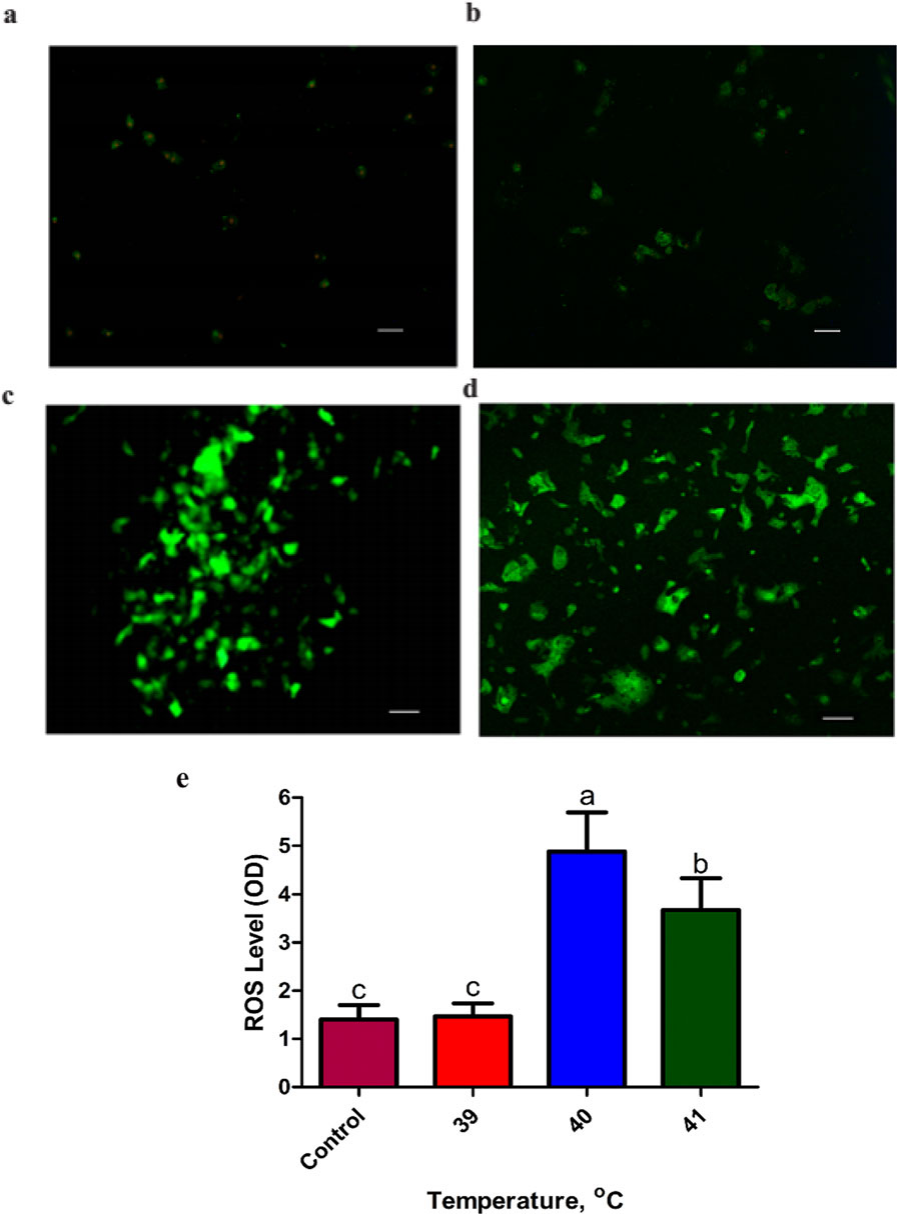
Heat stress enhanced intracellular ROS accumulation in bovine granulosa cells: Fluorescent photomicrographs of bovine granulosa cells (bGCs) stained with 2′,7′-dichlorofluorescin diacetate (H_2_DCFDA) were shown control (38 °C) (**a**) and heat stress (39, 40 and 41 °C) (**b**, **c**, **d**, respectively). The images shown are representative of the three independent image acquisitions. **e** Quantitative analysis of relative fluorescence emission. Values are expressed as mean ± SEM of *n* = 3. Superscripts (a, b, c) show significant difference, *P* < 0.05

Following exposure to heat stress at 40 °C, an increasing level of intracellular ROS accumulation was observed in granulosa cells compared to other cell cultured groups (Fig. 5c). However, there were no significant differences in ROS accumulation at 39 °C (Fig. 5a, b, e). Furthermore, the relative fluorescence emissions were significantly higher (*P* < 0.05) when bGCs were exposed to 41 °C than the control group but lower than 40 °C (Fig. 5d).

### RNA sequencing data analysis for identifying differentially expressed genes among three groups (control vs. 39 °C, control vs. 40 °C and control vs. 41 °C)

**Fig. 6:**
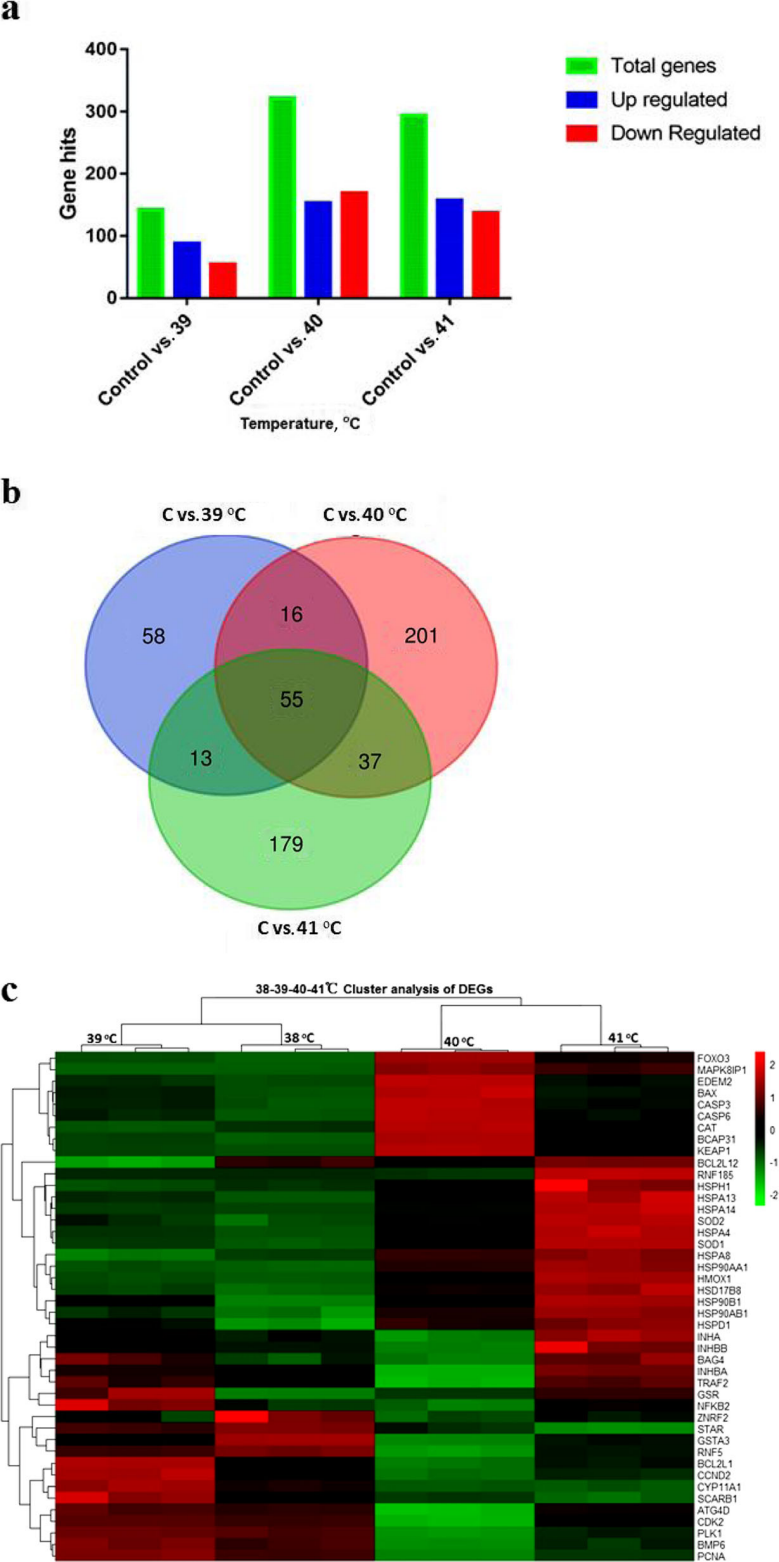
RNA sequencing data Analysis for identifying differentially expressed genes among three groups (Control vs. 39 °C, Control vs. 40 °C and Control vs. 41 °C): DEGs in different comparisons in bGCs. **a** Graphical representation of significant DEGs disclosed among three comparison groups of bovine granulosa cells cultured under different intensities of heat stress. **b** Venn diagrams shows overlapping DEGs after heat stress among three comparisons. **c**. Heatmap of top 45 differentially expressed granulosa cell genes in heat stressed groups with FC > 2, *P* < 0.05. Red corresponds to up-regulated gene product, and green corresponds to down-regulated gene product. Each differentially expressed gene is represented by a single row, and each heat treatment group is represented by a single column

In this study, an attempt was made to obtain a global picture of the *in-vitro* heat stress response by investigating the transcriptome profile of bGCs. Differentially expressed genes (DEGs) of bGCs were identified via RNA-Seq to analyze genome-wide transcriptional expression differences among the three groups. Under the criteria of |fold change| (|FC|) > 1.5 and *P* < 0.05, 142 DEGs, including 88 (61.9%) up-regulated and 54 (38%) down-regulated were identified by comparing control versus (vs.) 39 °C group. Similarly, for Control vs. 40 °C, a total of 321 DEGs with 153 (47.6%) up and 169 (52.6%) down-regulated were reported. During the comparison of control versus 41 °C, 294 significantly DEGs were detected containing 157 (53.4%) up and 137 (46.5%) down-regulated genes (Additional file 1, Table 1, Fig. 6a). Results revealed the highest number of DEGs in Control vs. 40 °C, whereas the least DEG number was detected in control vs. 39 °C group. These results indicate a strong inducement of genes in 40 °C bGCs cultured group.

**Table 1:** Number of differentially expressed genes (DEGs) disclosed in three comparisons of bovine granulosa cells (bGCs) after heat stress

Groups	Total genes	Up regulated	Down regulated	Criteria
Control vs. 39 °C	142	88	54	FC > 1.5, *P* < 0.05
Control vs. 40 °C	321	153	169	FC > 1.5, *P* < 0.05
Control vs. 41 °C	294	157	137	FC > 1.5, *P* < 0.05

### Heat stress resulted in the activation of differentially expressed heat shock factors, apoptotic, steroidogenic and oxidative stress-related genes

Among the several hundred genes induced or repressed as a result of *in-vitro* heat stress, an effort was made to filter out genes related to; heat shock protein family, apoptosis; steroidogenesis and oxidative stress (Table 2). A heatmap and hierarchical clustering of the top 45 significant (*P* < 0.05) differentially expressed genes with |FC| > 1.5 and *P* < 0.05 demonstrates the relatedness of samples, as shown in Fig. 6c.

**Table 2:**
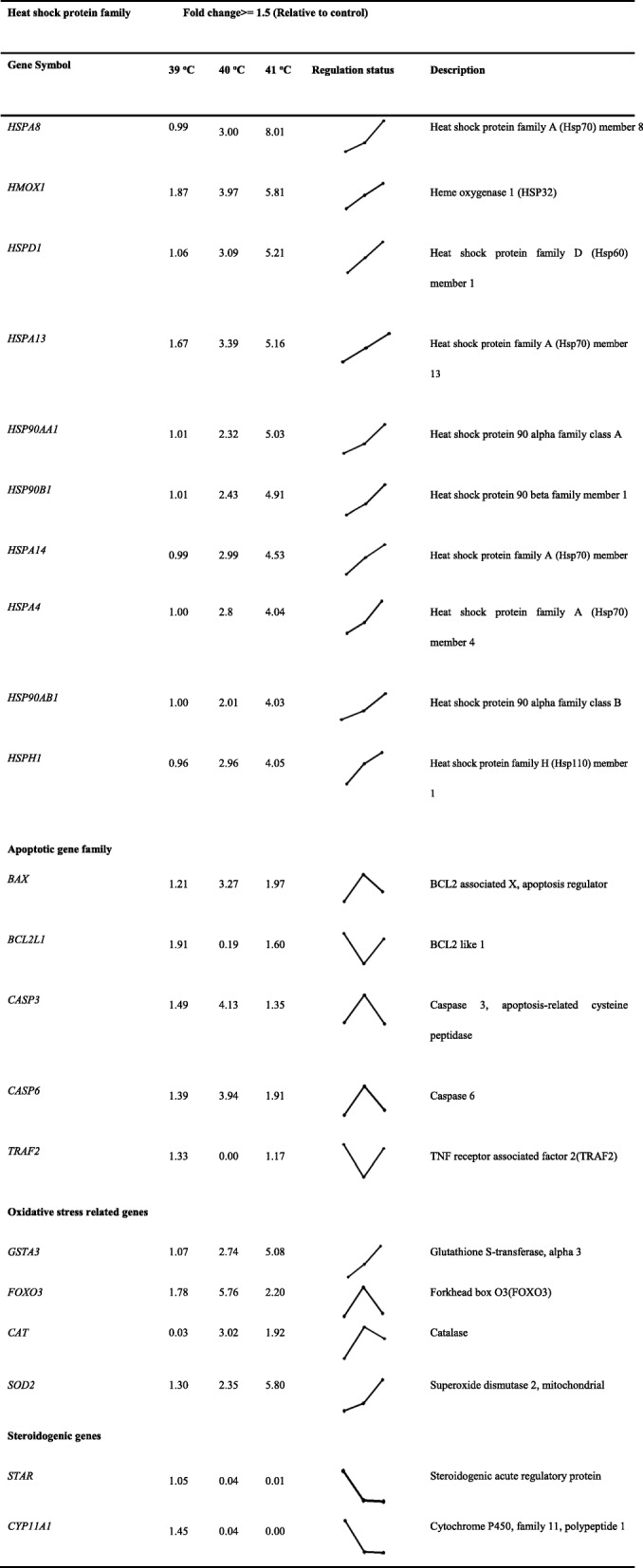
List of genes classified in major functional categories during post heat stress (relative to Control) in bGCs

### Pathway analysis of differentially expressed genes in response to heat stress

For an enhanced understanding of signaling pathways regulated by the heat treatments, the identified DEGs in the three comparisons were subjected to pathway analysis using KEGG.

### Control vs. 39 °C cultured group

**Fig. 7:**
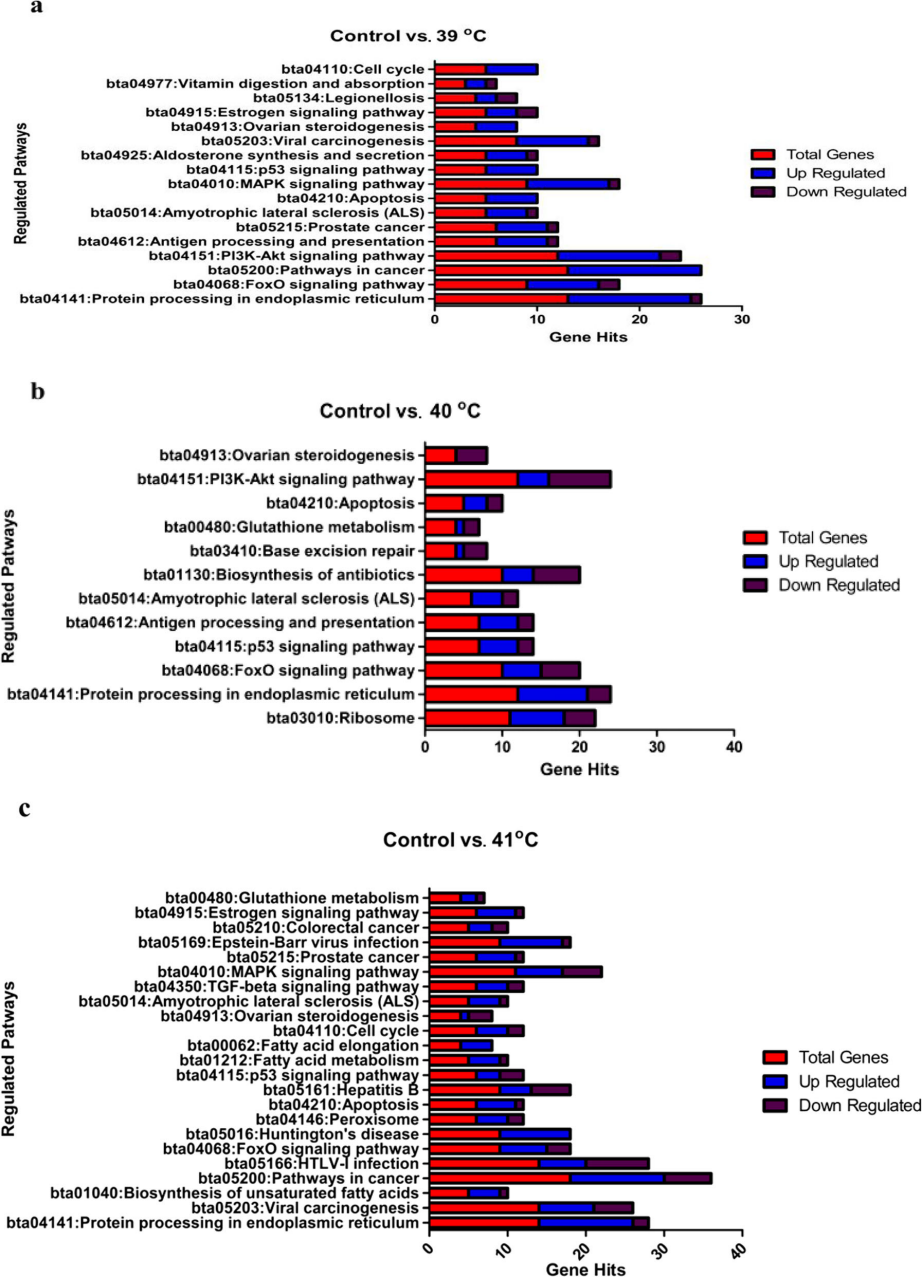
Pathway analysis of differentially expressed genes among three groups in response to heat stress: Enriched gene pathways in granulosa cells along all comparison of Control vs. 39, 40 and 41 °C cultured groups. Only significantly (*P* < 0.05) regulated pathways with up and down genes were shown (**a**, **b**, **c**)

A total of 25 canonical pathways enriched by genes differentially expressed in granulosa cells in this comparison (Additional file 2), of which 18 were significantly (*P* < 0.05) regulated (Fig. 7a, Table 3) while the rest of the eight did not meet the criteria for significance (*P* < 0.05). The number of up and down-regulated DEGs involved in these 18 KEGG pathways are presented in Fig. 7a. Furthermore, among significantly regulated pathways, 15 were directly related to the qualitative traits of bGCs under heat stress and the genes distributed in each pathway are enlisted in (Additional file 2).

**Table 3:** The DEGs and biological pathways regulated in treatment groups (Control vs. 39 °C, Control vs. 40 °C, Control vs. 41 °C)

Pathway name	Gene count	*P*-value	Gene name
Control vs. 39 °C			
Protein processing in endoplasmic reticulum	13	1.23E-07	*HSP90AB1, TRAF2, HSPH1, ATF4, HSP90B1, HSP90AA1, RNF5, BAX, RNF185, EDEM2, EIF2AK4, HSPA8, BCAP31*
FoxO signaling pathway	9	5.74E-05	*PLK1, CCND2, MDM2, CAT, FOXO3, G6PC3, CDK2, SOD2*
Pathways in cancer	13	7.19E-04	*HSP90AB1, TRAF2, CASP3, HSP90B1, HSP90AA1, BAX, GNB5, MDM2, BCL2L1, NFKB2, FGF12, CRK, CDK2*
PI3K-Akt signaling pathway	12	7.77E-04	*HSP90AB1, ATF4, HSP90B1, HSP90AA1, CCND2, GNB5, MDM2, FOXO3, BCL2L1, FGF12, G6PC3, CDK2*
Antigen processing and presentation	6	9.96E-04	*HSP90AB1, HSP90AA1, NFYC, IFI30, HSPA4, HSPA8*
bta05215:Prostate cancer	6	1.80E-03	*HSP90AB1, ATF4, HSP90B1, HSP90AA1, MDM2, CDK2*
Amyotrophic lateral sclerosis (ALS)	5	2.20E-03	*CASP3, BAX, CAT, BCL2L1, SOD1*
Apoptosis	5	3.70E-03	*CASP6, TRAF2, CASP3, BAX, BCL2L1*
MAPK signaling pathway	9	4.70E-03	*TRAF2, CASP3, ATF4, MAPK8IP1, NFKB2, FGF12, CRK, HSPA8, PPP5C*
p53 signaling pathway	5	6.00E-03	*CASP3, CCND2, BAX, MDM2, CDK2*
Aldosterone synthesis and secretion	5	8.70E-03	*ATF4, CYP11A1, STAR, SCARB1, ATF1*
Viral carcinogenesis	8	0.010	*TRAF2, CASP3, ATF4, CCND2, BAX, MDM2, NFKB2, CDK2*
Ovarian steroidogenesis	4	0.014	*CYP11A1, STAR, SCARB1, BMP6*
Estrogen signaling pathway	5	0.018	*HSP90AB1, ATF4, HSP90B1, HSP90AA1, HSPA8*
Legionellosis	4	0.020	*CASP3, HSPD1, NFKB2, HSPA8*
Vitamin digestion and absorption	3	0.021	*BTD, ABCC1, SCARB1*
Cell cycle	5	0.039	*PLK1, CCND2, PCNA, MDM2, CDK2*
Epstein-Barr virus infection	6	0.047	*TRAF2, MDM2, NFKB2, CDK2, EIF2AK4, HSPA8*
Control vs. 40 °C			
Ribosome	11	2.18E-04	*RPS26, MRPL23, MRPL21, MRPL15, RPLP0, RPL15, MRPL36, RPS11, MRPL33, UBA52, RPS24*
Protein processing in endoplasmic reticulum	12	3.51E-04	*HSP90AB1, TRAF2, HSPH1, HSP90B1, HSP90AA1, EIF2AK1, RNF5, BAX, RNF185, EDEM2, HSPA8, BCAP31*
FoxO signaling pathway	10	8.75E-04	*HRAS, PLK1, CCND2, PRKAB1, CAT, FOXO3, CCNG2, CDK2, SOD2*
p53 signaling pathway	7	0.002	*CASP3, CCND2, BAX, TSC2, RPRM, CCNG2, CDK2*
Antigen processing and presentation	7	0.002	*HSP90AB1, CTSL, HSP90AA1, NFYC, HSPA4, RFXANK, HSPA8*
Amyotrophic lateral sclerosis (ALS)	6	0.003	*CASP3, BAX, TOMM40L, CAT, BCL2L1, SOD1*
Biosynthesis of antibiotics	10	0.016	*SC5D, UAP1, IDH3G, PAFAH2, PGM1, RCE1, CAT, UAP1L1, PAICS, OAT*
Base excision repair	4	0.024	*LIG3, PCNA, OGG1, APEX1*
Glutathione metabolism	3	0.026	*GSS, GSR, GSTA3*
Apoptosis	5	0.029	*CASP6, TRAF2, CASP3, BAX, BCL2L1*
PI3K-Akt signaling pathway	12	0.035	*HSP90AB1, HRAS, HSP90B1, HSP90AA1, CCND2, TSC2, GNG2, FOXO3, BCL2L1, FGF12, CDK2, GHR*
Ovarian steroidogenesis	4	0.038	*CYP11A1, STAR, SCARB1, BMP6*
Control vs 41 °C			
Protein processing in endoplasmic reticulum	14	1.40E-05	*HSP90AB1, TRAF2, HSP90AA1, RNF185, EDEM2, BCAP31, HSPH1, HSP90B1, EIF2AK1, DNAJB11, RNF5, BAX, EIF2AK2, HSPA8*
Viral carcinogenesis	14	3.92E-04	*TRAF2, CASP3, HDAC1, CCND2, DDB1, BAX, CREBBP, RHOA, PIK3CA, NFKB2, EIF2AK2, SRF, CDK2, SCRIB*
Biosynthesis of unsaturated fatty acids	5	9.11E-04	*PECR, ACOT7, ELOVL5, HACD2, FADS2*
Pathways in cancer	18	0.001	*HSP90AB1, DVL2, TRAF2, HSP90AA1, EPAS1, CREBBP, BCL2L1, KIT, NFKB2, CDK2, VEGFC, FOS, CASP3, HSP90B1, HDAC1, BAX, RHOA, PIK3CA*
HTLV-I infection	14	0.001	*DVL2, TSPO, MRAS, CREBBP, NFKB2, BCL2L1, SRF, FOS, CCND2, BAX, PCNA, RRAS, PIK3CA, NFATC3*
FoxO signaling pathway	9	0.003	*PLK1, CCND2, CREBBP, PIK3CA, CAT, FOXO3, CDK2, SOD2*
Huntington's disease	9	0.003	*CASP3, HDAC1, BAX, CREBBP, NDUFV2, NDUFS3, SOD1, SOD2*
Peroxisome	6	0.004	*PECR, PEX2, MPV17, CAT, SOD1, SOD2*
Apoptosis	6	0.005	*CASP6, TRAF2, CASP3, BAX, PIK3CA, BCL2L1*
Hepatitis B	9	0.006	*FOS, CASP3, DDB1, BAX, CREBBP, PCNA, PIK3CA, NFATC3, CDK2*
p53 signaling pathway	6	0.010	*CASP3, CCND2, CD82, BAX, CDK2*
Fatty acid metabolism	5	0.011	*PECR, ELOVL5, HACD2, FADS2, HADH*
Fatty acid elongation	4	0.011	*ACOT7, ELOVL5, HACD2, HADH*
Cell cycle	6	0.013	*HDAC1, PLK1, CCND2, CREBBP, PCNA, CDK2*
Ovarian steroidogenesis	4	0.015	*CYP11A1, STAR, SCARB1, BMP6*
Amyotrophic lateral sclerosis (ALS)	5	0.017	*CASP3, BAX, CAT, BCL2L1, SOD1*
TGF-beta signaling pathway	6	0.018	*INHBB, INHBA, CREBBP, RHOA, TGIF2, BMP6*
MAPK signaling pathway	11	0.021	*TRAF2, FOS, CASP3, MRAS, PTPRR, RRAS, MAPK8IP1, NFKB2, NFATC3, SRF, HSPA8*
Prostate cancer	6	0.021	*HSP90AB1, HSP90B1, HSP90AA1, CREBBP, PIK3CA, CDK2*
Epstein-Barr virus infection	9	0.026	*TRAF2, EIF2AK1, HDAC1, CREBBP, PIK3CA, NFKB2, EIF2AK2, CDK2, HSPA8*
Colorectal cancer	5	0.0339	*FOS, CASP3, BAX, RHOA, PIK3CA*
Estrogen signaling pathway	6	0.0354	*HSP90AB1, FOS, HSP90B1, HSP90AA1, PIK3CA, HSPA8*
Glutathione metabolism	3	0.0386	GSR, GSTA3, G6PD

### Control vs. 40 °C cultured group

Total of 18 canonical pathways was enriched in response to heat stress; out of them, 12 were significantly (*P* < 0.05) regulated (Additional file 2, Table 3). The 13 pathways which have key roles in the apoptosis, oxidative stress, antioxidant and steroidogenesis regulation of the bGCs were selected and shown in Fig.7b based on up and down-regulated genes. Between the comparisons of Control vs. 39 °C and Control vs. 40 °C, seven commonly shared pathways were reported. Furthermore, our findings concealed that most of the DEGs among these pathways were up-regulated (Additional file 2). With the increase in heat stress in Control vs. 40 °C comparison. Glutathione metabolism pathways were up-regulated to combat stress by regulating antioxidant genes (*SOD1, SOD2*, etc.) (Fig. 7b).

### Control vs. 41 °C cultured group

Out of 28 canonical KEGG enriched pathways in control vs. 41 °C comparison, 23 reached a significant level (*P* < 0.05) and are shown based on up and down-regulated genes (Additional file 2, Fig. 7c, Table 3). Furthermore, 14 pathways were involved in the regulation of apoptosis, oxidative stress, antioxidant, and steroidogenesis regulation of the bGCs under heat stress (Additional file 2). Taking all comparisons, it was found that five pathways (Protein processing in the endoplasmic reticulum, FoxO signaling pathway, Apoptosis, p53 signaling pathway, and Pathways in cancer) were shared in all the three comparisons.

### Commonly shared genes among all the pathways of the three comparisons

The total of 142, 321 and 294 significantly (*P* < 0.05) DEGs were documented in the three comparisons of Control vs. 39 °C, Control vs. 40 °C, and Control vs. 41 °C, respectively. Out of these DEGs, 55 genes were commonly shared among the three comparisons. Furthermore, 58, 201, and 179 DEGs were found to be unique genes for Control vs. 39 °C, Control vs. 40 °C, and Control vs. 41 °C, respectively (Additional file 3, Fig. 6b).

### Regulation of signaling pathways under heat stress affecting bGCs functions

**Fig. 8:**
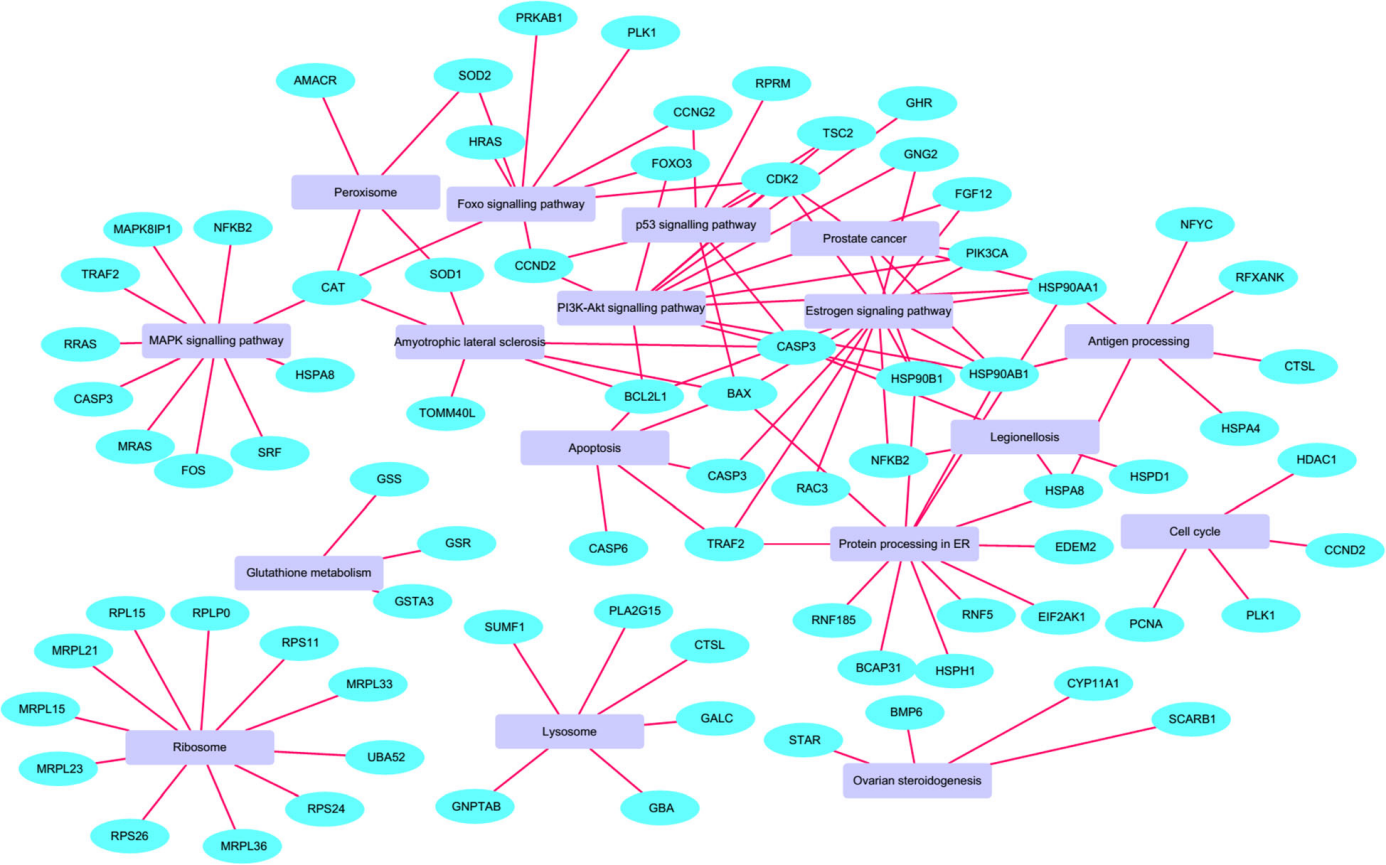
Regulation of signaling pathways under heat stress affecting bGCs functions: A network map of pathways significantly (*P* < 0.05) enriched after heat stress. The nodes are the pathways, and edges connect the genes involved in the pathway

Heat stress significantly regulated pathways are affecting bGCs physiological attributes i.e. promote the inhibition of cell growth, steroidogenesis, and induction of apoptosis by the accumulation of ROS, etc. These pathways include (MAPK signaling pathway, FoxO signaling pathway, Apoptosis, ovarian steroidogenesis, Protein processing in the endoplasmic reticulum, and Glutathione metabolism. Genes belonging to these canonical pathways were differentially expressed (Fig. 8) in response to HS.

### Functional annotation cluster and gene ontology analysis

Detailed annotation of molecular gene function, biological process and cellular distribution of differentially expressed genes (DEGs; > = 1.5 fold change) identified by gene ontology (GO) descriptions in response to heat stress in-vitro cultured bGCs was accomplished in order to explore biological significance.

### Control vs. 39 °C comparison

A total of 58, 24, and 16 biological processes (BP), cellular components (CC) and molecular functions (MF) respectively, were found to be affected by heat stress. However, the 35, 14, and 10 were enriched under BP, CC, and MF respectively revealed significant level (*P* < 0.05). A total of 142 DEGs (54 down-regulated, i.e., *CAT, MAPK8IP1, TMCO3* and 88 up-regulated, i.e., *BMP6, PRDX6,* and *HMOX1*) were involved in all GO terms. Among 35 biological processes, major molecular functions, i.e. oxidation-reduction process, regulation of the apoptotic process, cell redox homeostasis, cell development, regulation of MAPK cascade, ovarian follicle development, cholesterol transport, ATP binding, poly(A) RNA binding, and protein homodimerization activity were found to be associated with DEGs. The seven major cellular processes associated with DEGs were the nucleus, cytoplasm, extracellular exosome, cystole, nucleoplasm, and mitochondrion (Additional file 6). A complete list of GO terms and the genes involved in them are given in the (Additional file 4).

### Control vs. 40 °C comparison

GO analysis was conducted on DEGs (≥ 1.5 fold change) that summarized the major GO terms influenced by heat stress in granulosa cells. DEGs regulated a total of 52 BP, 28 CC, and 15 MF in Control vs. 40 °C treated group. Out of them, 40, 21, and 09 showed a significant difference (*P* < 0.05) for BP, CC, and MF, respectively (Additional file 6). Total of 321 DEGs (169 down-regulated, i.e., *PCNA, BAG4, BMP6, INHA* and 153 up-regulated, i.e., *CASP3, FOXO3, KEAP1*) were significantly involved in all GO terms. Out of 51 biological function processes, major gene portions were enriched in regulation of the apoptotic process, regulation of cell cycle, protein folding, DNA repair, negative regulation of cholesterol biosynthetic process and superoxide metabolic process (Additional file 6). Similarly, the GO terms for MF included structural constituent of ribosome, protein homodimerization activity, RNA binding, cysteine-type endopeptidase activity, etc. (Additional file 6). Moreover, CC related GO terms were cytoplasm, nucleus, extracellular exosome, mitochondrion, endoplasmic reticulum, etc. (Additional file 6). A complete set of GO terms and the DEGs involved are given in the (Additional file 4).

### Control vs. 41 °C comparison

A total of 294 DEGs (137 down-regulated, i.e., *CYP11A1, STAR, SCARB1*and 157 up-regulated, i.e., *SOD2, HSP90AA1, HSPD1*) in heat stressed bGCs in the comparison of Control vs. 41 °C were also assigned with GO terms. A wide range of GO categories have been identified for the biological process, including cellular response to oxidative stress, negative regulation of the apoptotic process, MAPK cascade regulation, glutathione metabolic process, cellular response to reactive oxygen species, etc.. GO terms for molecular functions were also identified for 294 DEGs that were commonly over expressed at 41 °C. They comprise poly(A) RNA binding, superoxide dismutase activity, protein binding, protein homodimerization activity, etc. (Additional file 6). Similarly, GO terms for cellular components, i.e. cytoplasm, extracellular exosome, Bcl-2 family protein complex, mitochondrion, endoplasmic reticulum, transcription factor complex, etc. were affected (Additional file 6). Data set for all GO terms are shown in the (Additional file 4).

### Protein-protein interaction (PPI) networks of DEGs significantly enriched pathways associated with bGCs functions under heat stress

**Fig. 9:**
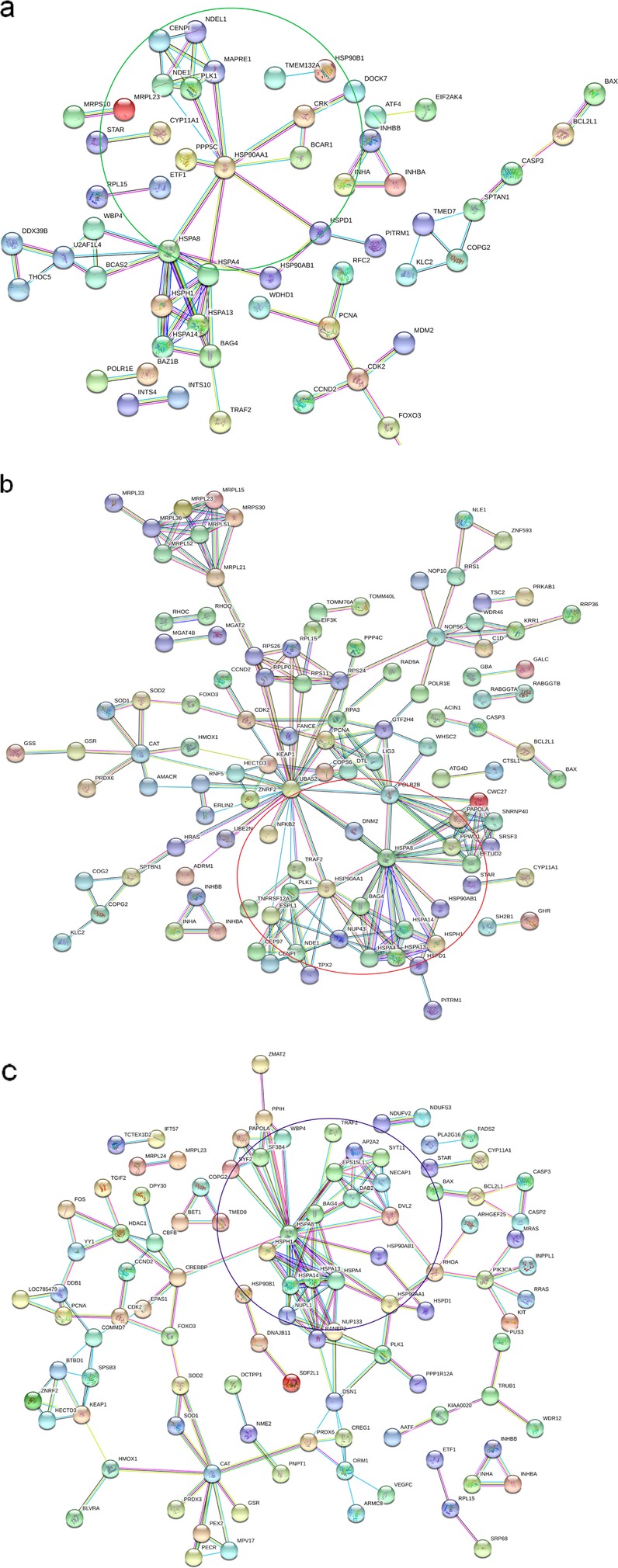
Protein-protein interaction (PPI) networks of DEGs significantly enriched pathways associated with bGCs functions under heat stress: Protein-protein interaction (PPI) networks in the comparison of Control vs. 39 °C (**a**) Control vs. 40 °C (**b**) and Control vs. 41 °C (**c**). Various color lines represent seven types of evidence used in predicting associations. Red line: fusion evidence; blue line: co-occurrence evidence; yellow line: text mining evidence; green line: neighborhood evidence; purple line: experimental evidence; light blue line: database evidence; and the black line: co-expression evidence

To get a better insight into the interconnection among DEGs regulated pathways under heat stress, STRING analysis with confidence (0.09) was used to draw an interaction network among the corresponding proteins of the DEGs across all comparisons (Control vs. 39 °C, Control vs. 40 °C and Control vs. 41 °C). The PPI interaction network analysis showed that compared with Control vs. 39 °C and Control vs. 41 °C, most of the proteins in Control vs. 40 °C are highly interconnected (Fig. 9a, b, c). Interestingly, the PPI in response to 39 °C treatment showed that *HSP90AA1, BCAR1, PPP5C, CRK, INHA, INHBA, INHBB, DOCK7,* and *MAPRE1* distributed in the central parts. While in 40 °C and 41 °C treated GCs cultured group, PPI network analysis revealed that the HSPs, cochaperones (*HSP90AA1, HSP90AB1, HSPA13, HSPA4, HSPA8, HSPA14, HSPD1, HSPH1, BAG4, NUP43, TRAF2, PLK1*, etc.) occupied central position and were strongly related and mostly co-expressed.

### Validation of RNA-Seq results by RT-qPCR

To confirm changes in the expression of genes identified in RNA-Seq results, quantitative reverse transcription polymerase chain reaction (RT-qPCR) analyses of 15 representative genes were performed on the same samples (Additional file 5). Gene expression profiling of granulosa cells showed that some HSP family-related genes were active during cellular heat response (Table 2). The expressions of HSP family genes such as *HSPA13*, *HMOX1*, apoptotic-related genes (*CASP3*, *BAX* and *BCL2L1*), steroidogenic genes (*CYP11A1, STAR*) antioxidant activity-related genes (*SOD2, CAT, GSTA3*) and genes related to oxidative stress (*FOXO3* and *MAPK8IP1*) were significantly (*P* < 0.05) regulated across all heat-treated granulosa cells compared to the control group. The results showed that all the genes had similar expression trends as detected in the RNA-Seq. This consistency between RT-qPCR and RNA-Seq revealed the reliability of our RNA-Seq data (Additional file 6).

## Discussion

**Fig. 10:**
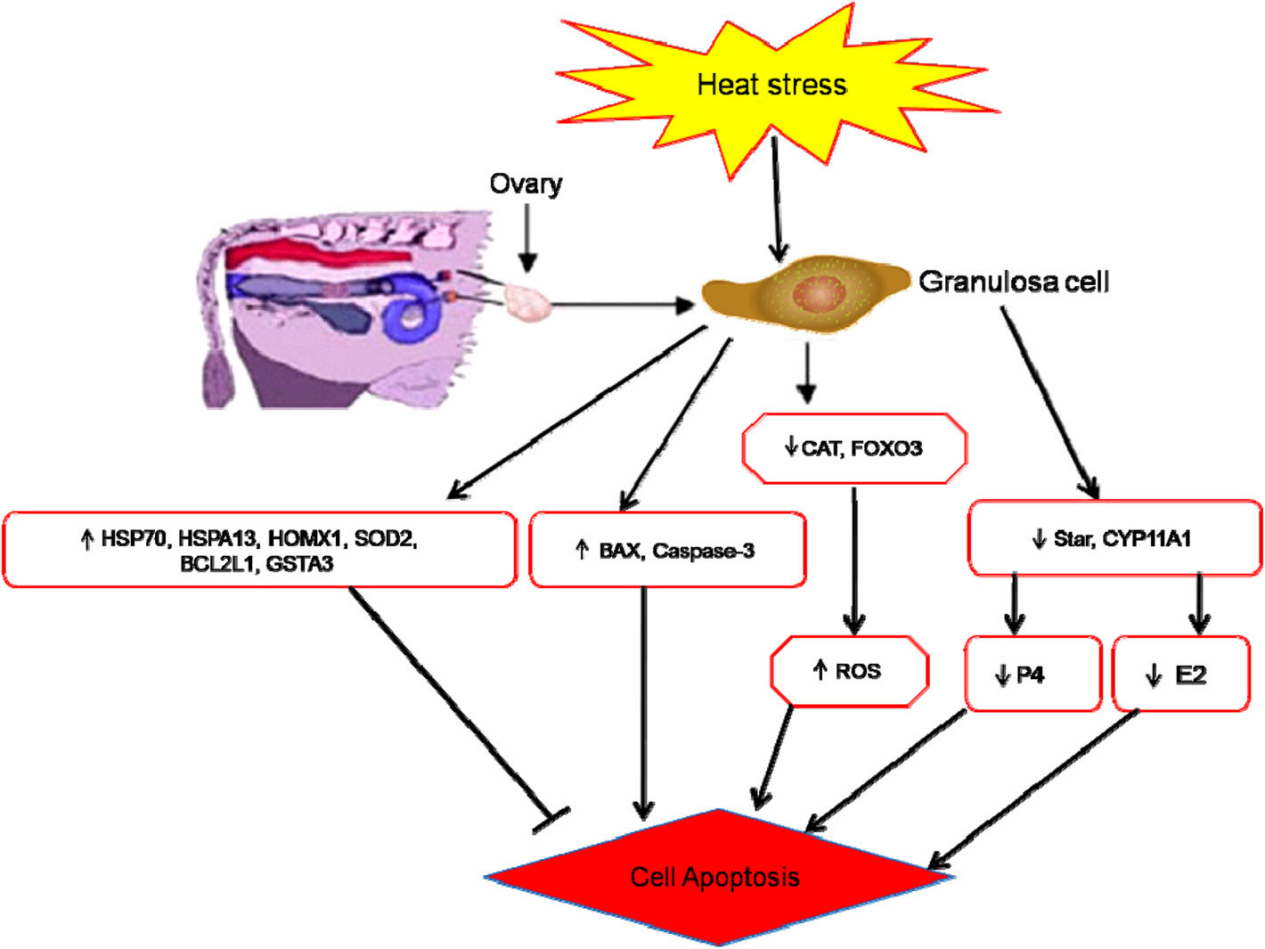
Research review: Mechanisms of regulating heat stress response related to follicular function within bovine ovary. Upregulated genes caspase-3, *SOD*, *BCL-2, BAX,* and HSPs (*HSP70, HSPA13, HMOX1*) were involved in the regulating mechanism of bGCs via induced or inhibited cell apoptosis. Under heat stress, Down-regulated genes *CAT*, *FOXO3* were involved in production of reactive oxygen species (ROS). Likewise, down-regulation of *STAR*, and *CYP11A1* were involved in the secretion of E_2_ and P_4_. Moreover, the decline of E_2_, and enhancing of ROS in turn, might enhance the possibility of GC apoptosis and follicle function

Environmental factors, particularly temperature, have a significant impact on animal breeding and reproduction [22]. Heat stress can be defined as a condition that occurs when an animal can not dissipate body heat adequately to maintain thermal equilibrium [23, 24]. Heat is proteotoxic stress and causes denatured proteins that, by forming aggregates, can become cytotoxic [25]. The ovarian follicle’s granulosa cells play a crucial role in oocyte nourishing, secreting hormones that create functional bidirectional crosstalk with the oocyte [26]. A brief overview of the current study and mechanisms of regulating heat stress response related to follicular function within the bovine ovary is shown in Fig. 10.

In the present study, bGCs were exposed to different levels of *in-vitro* heat stress and found that heat stress involves compromising the physiological functions of bGCs by increasing intracellular accumulation of ROS, inducing apoptosis and reducing the synthesis of E2 and P4 [7, 9, 15]. For more understanding, we conducted the transcriptomic study of *in-vitro* cultured bGCs exposed to heat stress at 39, 40, and 41 °C. Amongst the several hundred genes induced or repressed due to heat stress *in-vitro*, an attempt was taken to screen out genes associated with, heat shock protein family, apoptosis, steroidogenesis and oxidative stress (Table 2). As expected, the whole set of genes of heat shock family viz., *HSPA8, HSPA14, HSP90AA1, HMOX* 1, etc. were up-regulated in bGCs at most of the heat stress points. The expression of these genes was more at 41 °C of heat stress as compared to other treated groups (39, 40 °C). Our findings are supported by previous studies that led to the induction of HSP genes by heat stress [27, 28]. Similar to our study, HSPs induction was reported in various cell/tissue types such as leukocytes/lymphocytes [29–31], bovine endometrial tissue, bovine conceptuses [32, 33], bovine granulosa cells [34] bovine MECs [22], buffalo lymphocytes [35] due to heat stress. It has been reported that heat stress causes an increase in HSPs in virtually all the vertebrates, including mice [36, 37] domestic goats [38], humans [39, 40], juvenile Hamadryads baboons [41], common carp [42], domestic chickens [43–46] and domestic turkey [47]. Our results showed an increased accumulation of inducible HSP70 in heat stressed groups at both the protein and mRNA levels, thus supporting the idea that HSP70 can act as a reliable thermal stress biomarker [42, 48]. Likewise, several apoptosis-related genes like *BCL2* associated X, apoptosis regulator (*BAX*), caspase 3, apoptosis-related cysteine peptidase (*CASP3*) and (*CASP6*), etc. were also found to be significantly (*P* < 0.05) up-regulated under heat stress that signal through the apoptosis signaling pathway. Up-regulation of apoptotic genes could lead to disruption of the potential of mitochondrion transmembrane, resulting in the release of cytochrome c leading to apoptosis induction [49]. Data on the induced expression of apoptotic genes at 40 °C suggest that the cellular mechanism may not provide protection for bGCs against heat-induced apoptosis while the rate of apoptosis decreased at 41 °C of heat stress due to over-expression of *HSP70, HSP90* and *HSP60* protein levels likely helped bGCs activate self-protection mechanisms and cope with hyperthermia through clearance of damaged proteins. Our results are in line with some previous reports where the MAPK mediated induction of *HSP70* at high temperature could play a crucial role in inhibiting caspase-3 and *BAX* activation [50, 51]. Therefore, we suggest that the induction of *HSP70* occurs to reduce apoptosis of granulosa cells induced by heat stress. This is a first study that unveiled the effect of heat stress with different intensities on apoptosis-related gene expression and on the cellular defensive mechanism in bGCs.

Heat stress results in intracellular ROS accumulation, causing oxidative stress [52] and apoptosis [53], which subsequently lead to a decline in fertility [54, 55]. Moreover, the current study also shows for the first time induction of ROS at different intensities of heat stress in bGCs. Compared to the control, significant (*P* < 0.05) ROS accumulation was evident at 40 °C and 41 °C of heat stress, but at 39 °C the ROS induction was not significant. We found a decline in ROS levels in bGCs by increasing treatment temperature from 40 °C to 41 °C. This may be due to the cells being able to activate their antioxidant systems at a higher temperature of 41 °C, by regulating genes, i.e. superoxide dismutase 1, 2 (*SOD1*, *SOD2,*), glutathione-disulfide reductase (*GSR*) and glutathione S-transferase, alpha 3 (*GSTA3*) to protect cells against oxidative stress. In addition, the high expression of HMOX1 gene was observed in the culture of human melanoma cells, confirming the induction of cellular oxidative stress during harmful insults [56]. Similar to our results, the activation of forkhead box O3 (*FoxO3*) and kelch-like ECH associated protein 1 (*KEAP1*) under heat stress protects cells from oxidative stress by up-regulating antioxidant enzymes superoxide dismutase 2 (*SOD2*) and catalase (*CAT*) [57–59]. In *Saccharomyces cerevisiae* and quail, genes from the glutathione peroxidase family were also shown to be induced under heat stress [60, 61]. Based on these facts, it is reasonable to suggest that the up-regulation expression of *SOD2* and *CAT* may inhibit ROS biosynthesis through the regulation of *KEAP1*and *FOXO3* in ovarian granulosa cells.

Moreover, the regulation of genes related to steroidogenesis, i.e. steroidogenic acute regulatory protein (*STAR*) and Cytochrome P450, family 11, subfamily A, polypeptide 1 (*CYP11A1*) was also affected by heat shock. Previously it was reported that heat stress could inhibit estradiol biosynthesis in bGCs and impair hormone balance [62]. Positive regulation of P450 aromatase family genes (*CYP11A1*) in the ovarian follicle promotes estrogen biosynthesis [63]. In our study, the mRNA expression of *CYP11A1* decreased in GCs by down-regulation of ovarian steroidogenesis signaling pathway after heat treatment that resulted in a decreased level of E_2_ in the culture medium. Based on this confirmation, we can postulate that the down-regulation of *CYP11A1* may inhibit estrogen biosynthesis in ovarian granulosa cells. In addition, progesterone is also one of the fundamental steroid hormones for bovine estrous cycle regulation, and its biosynthesis is attributed to the increased expression of *STAR* and *CYP11A1* [64–66]. Previously it was reported that under heat stress, the mRNA expression of *CYP11A1* and *STAR* decreased, but the P4 level has no significant (*P* < 0.05) difference between the control and heat treatment group [9]. An over-secretion of ovarian hormones in porcine ovarian granulosa cells were reported under high temperature [67]. Our findings are in line with the previous studies where heat stress attenuates estrogenic activity in rat granulosa cells by diminishing the expression of gonadotropin receptor [68]. We also found a lower mRNA expression of the genes *CYP11A1* and *STAR* in heat-treated groups. This is the first study to establish the impact of different intensities of thermal stress on the synthesis of steroid hormones and gene expression profile in bGCs. These findings have provided evidence to suggest the varied expression profile in bGCs during heat stress of apoptotic, steroidogenesis and oxidative stress-related genes. In the current study, the RT-qPCR analysis thus validated the transcriptional expression profile of HSPs, apoptotic genes, steroidogenesis, and oxidative stress-related genes as observed by RNA-Seq analysis. Our research can further be extended to understand bovine oocyte modulation and embryo development in response to the environmental heat load.

## Conclusion

In the present study, we demonstrated for the first time a worthy strategy to characterize the cellular and transcriptomic adaptation of bovine granulosa cells to different heat stress intensities (39 °C, 40 °C and 41 °C) *in-vitro*. Furthermore, our data suggested that 40 °C heat treatment is comparatively detrimental for bovine granulosa cell functions. The study identified several heat-responsive genes from different functional classes and their associated pathways related to heat stress chaperons, cell death, and apoptosis, hormonal synthesis, oxidative stress, etc. known to be affected by heat stress. The results infer that these genes and pathways reported in the present study could be useful candidates/indicators for heat stress research in dairy cattle. Moreover, the established model of bGCs to heat stress in the current study provides an appropriate platform to understand the mechanism of how heat-stressed bGCs can affect the quality of oocytes and developing embryo.

## Supplementary information

**Additional file 1:** List of significant differentially expressed genes (DEGs) along all treatments.

**Additional file 2:** Regulated Pathways under all treatments of heat stress.

**Additional file 3:** Commonly shared DEGs across all treatments.

**Additional file 4:** Gene Ontology terms regulated across all treatments.

**Additional file 5:** Table of gene primers for RT-qPCR.

**Additional file 6:** Figures.

**Additional file 7:** Covering letter.

## Data Availability

All data generated or analyzed during this study available from the corresponding authors on reasonable request.

## Abbreviations

bGC: Bovine Granulosa cell
BP: Biological Process
CC: Cellular Components
COC: Cumulus Oocyte Complex
DEGs: Differentially Expressed Genes
DPBS: Dulbecco’s Phosphate-Buffered Saline
E_2_: Estradiol
GO: Gene Ontology
HS: Heat Stress
HSPs: Heat Shock Proteins
KEGG: Kyoto Encyclopedia of Genes and Genomes
MF: Molecular Functions
P_4_: Progesterone
PPI: Protein-Protein Interaction
RNA-Seq: RNA sequencing
ROS: Reactive Oxygen Species
RT-qPCR: Quantitative Reverse Transcription Polymerase Chain Reaction
THI: Temperature Humidity Index

## References

[1] Su YQ, Wu X, O’Brien MJ, Pendola FL, Denegre JN, Matzuk MM (2004). Synergistic roles of BMP15 and GDF9 in the development and function of the oocyte-cumulus cell complex in mice: genetic evidence for an oocyte-granulosa cell regulatory loop. Dev Biol.

[2] Petro EM, Leroy JL, Van Cruchten SJ, Covaci A, Jorssen EP, Bols PE (2012). Endocrine disruptors and female fertility: focus on (bovine) ovarian follicular physiology. Theriogenology.

[3] Eppig JJ (2001). Oocyte control of ovarian follicular development and function in mammals. Reproduction.

[4] Bettaieb A, Averill-Bates DA (2008). Thermotolerance induced at a fever temperature of 40 degrees C protects cells against hyperthermia-induced apoptosis mediated by death receptor signalling. Biochem Cell Biol.

[5] Wegner K, Lambertz C, Das G, Reiner G, Gauly M (2016). Effects of temperature and temperature-humidity index on the reproductive performance of sows during summer months under a temperate climate. Anim Sci J.

[6] Hansen PJ, Arechiga CF (1999). Strategies for managing reproduction in the heat-stressed dairy cow. J Anim Sci.

[7] Roth Z, Arav A, Bor A, Zeron Y, Braw-Tal R, Wolfenson D (2001). Improvement of quality of oocytes collected in the autumn by enhanced removal of impaired follicles from previously heat-stressed cows. Reprod.

[8] Sirotkin AV (2010). Effect of two types of stress (heat shock/high temperature and malnutrition/serum deprivation) on porcine ovarian cell functions and their response to hormones. J Exp Biol.

[9] Li J, Gao H, Tian Z, Wu Y, Wang Y, Fang Y (2016). Effects of chronic heat stress on granulosa cell apoptosis and follicular atresia in mouse ovary. J Anim Sci Biotechnol.

[10] Calderwood SK, Stevenson MA, Murshid A (2012). Heat shock proteins, autoimmunity, and cancer treatment. J Rheumatol Autoimmune Dis.

[11] Hou CH, Lin FL, Hou SM, Liu JF (2014). Hyperthermia induces apoptosis through endoplasmic reticulum and reactive oxygen species in human osteosarcoma cells. Int J Mol Sci.

[12] Samoylenko A, Hossain JA, Mennerich D, Kellokumpu S, Hiltunen JK, Kietzmann T (2013). Nutritional countermeasures targeting reactive oxygen species in cancer: from mechanisms to biomarkers and clinical evidence. Antioxid Redox Signal.

[13] Stetler RA, Gan Y, Zhang W, Liou AK, Gao Y, Cao G (2010). Heat shock proteins: cellular and molecular mechanisms in the central nervous system. Prog Neurobiol.

[14] Paul C, Teng S, Saunders PT (2009). A single, mild, transient scrotal heat stress causes hypoxia and oxidative stress in mouse testes, which induces germ cell death. Biol Reprod.

[15] Liu ZQ, Shen M, Wu WJ, Li BJ, Weng QN, Li M (2015). Expression of PUMA in Follicular Granulosa Cells Regulated by FoxO1 Activation During Oxidative Stress. Reprod Sci.

[16] Blondin P, Coenen K, Sirard MA (1997). The impact of reactive oxygen species on bovine sperm fertilizing ability and oocyte maturation. J Androl.

[17] Marshall A, Lukk M, Kutter C, Davies S, Alexander G, Odom DT (2013). Global gene expression profiling reveals SPINK1 as a potential hepatocellular carcinoma marker. PLoS One.

[18] Costa V, Aprile M, Esposito R, Ciccodicola A (2013). RNA-Seq and human complex diseases: recent accomplishments and future perspectives. Eur J Hum Genet.

[19] Pertea M, Daehwan K, Geo MP, Jeffrey TL, Steven LS (2016). Transcript-level expression analysis of RNA-seq experiments with HISAT, StringTie and Ballgown. Nat Protoc.

[20] Karla JH, David FA (2007). An oocentric view of folliculogenesis and embryogenesis. Reprod BioMed.

[21] Huang DW, Sherman BT, Lempicki RA (2009). Systematic and integrative analysis of large gene lists using DAVID bioinformatics resources. Nat Protoc.

[22] Collier RJ, Dahl GE, Van Baale MJ (2006). Major advances associated with environmental effects on dairy cattle. J Dairy Sci.

[23] Bernabucci U, Biffani S, Buggiotti L, Vitali A, Lacetera N, Nardone A (2014). The effects of heat stress in Italian Holstein dairy cattle. J Dairy Sci.

[24] Alves BG, Alves KA, Lúcio AC, Martins MC, Silva TH, Alves BG (2014). Ovarian activity and oocyte quality associated with the biochemical profile of serum and follicular fluid from Girolando dairy cows postpartum. Anim Reprod Sci.

[25] Liu T, Zhu S, Tang Q, Chen P, Yu Y, Tang S (2013). De novo assembly and characterization of transcriptome using Illumina paired-end sequencing and identification of CesAgene in ramie (Boehmeria niveaL.Gaud). BMC Genomics.

[26] Voronina E, Lovasco LA, Gyuris A, Baumgartner RA, Parlow AF, Freiman RN (2007). Ovarian granulosa cell survival and proliferation requires the gonad-selective TFIID subunit TAF4b. Dev Biolv.

[27] Heads RJ, Yellon DM, Latchman DS (1995). Differential cytoprotection against heat stress or hypoxia following expression of specific stress protein genes in myogenic cells. J Mol Cell Cardiol.

[28] Iwazawa M, Acosta TJ (2014). Effect of elevated temperatures on bovine corpus luteum function: expression of heat-shock protein 70, cell viability and production of progesterone and prostaglandins by cultured luteal cells. Anim Prod Sci.

[29] Agnew LA, Colditz IG (2008). Development of a method of measuring cellular stress in cattle and sheep. Vet Immuno Immunopathol.

[30] Dangi SS, Gupta M, Maurya D, Yadav VP, Panda RP, Singh G (2012). Expression profile of HSP genes during different seasons in goats (Capra hircus). Trop Anim Health Prod.

[31] Guerriero V, Raynes DA (1990). Synthesis of heat stress proteins in lymphocytes from livestock. J Anim Sci.

[32] Malayer JR, Hansen PJ, Buhi WC (1988). Effect of day of the oestrous cycle, side of the reproductive tract and heat shock on *in-vitro* protein secretion by bovine endometrium. J Reprod Fertil.

[33] Putney DJ, Malayer JR, Gross TS, Thatcher WW, Hansen PI, Drost M (1988). Heat stress-induced alterations in the synthesis and secretion of proteins and prostaglandins by cultured bovine conceptuses and uterine endometrium. Biol Reprod.

[34] Harada T, Koi H, Kubota T, Aso T (2004). Haem oxygenase augments porcine granulosa cell apoptosis *in vitro*. J Endocrinol.

[35] Mishra A, Hooda OK, Singh G, Meur SK (2011). Influence of induced heat stress on HSP70 in buffalo lymphocytes. J Anim Physiol Anim Nutr.

[36] Beck SC, Paidas CN, Tan H, Yang J, De MA (1995). Depressed expression of the inducible form of HSP70 (HSP 72) in brain and heart after *in vivo* heat shock. Am J Phys.

[37] Albers R, Bol M, Seinen W, Pieters R (1996). Stress proteins (HSP) and chemical-induced autoimmunity. Toxicol Appl Pharmacol.

[38] Meza-Herreraab CA, Martíneza L, Aréchigac C, Bañuelosc R, Rincónc RM, Urrutiab J (2005). Circannual identification and quantification of constitutive heat shock proteins (HSP 70) in goats. J App Anim Res.

[39] Hayashi Y, Iwai T, Toshio K, Tatsuya K, Kenzo O (1991). Translocation of hsp-70 and protein synthesis during continuous heating at mild temperatures in HeLa cells. Radiat Res.

[40] Kim D, Virginia W, Somji S, Garrett SH, Sens MA, Shukla D (2001). Expression of hsp 27, hsp 60, hsc 70, and hsp 70 by immortalized human proximal tubule cells (hk-2) following exposure to heat shock, sodium arsenite, or cadmium chloride. J Toxicol Environ Health A.

[41] Dehbi M, Baturcam E, Eldali A, Ahmed M, Kwaasi A, Chishti MA (2010). Hsp-72, a candidate prognostic indicator of heatstroke. Cell Stress Chaperones.

[42] Ferencz A, Juhasz R, Butnariu M, Deer AK, Varga IS, Nemcsok J (2010). Expression analysis of heat shock genes in the skin, spleen and blood of common carp (Cyprinuscarpio) after cadmium exposure and hypothermia. Acta Biol Hung.

[43] Givisiez PE, Ferro JA, Ferro MI, Kronka SN, Decuypere E, Macari M (1999). Hepatic concentration of heat shock protein 70 kD (Hsp70) in broilers subjected to different thermal treatments. Br Poult Sci.

[44] Hernandes R, Ferro JA, Gonzales E, Macari M, Bernal FEM, Ferro MIT (2002). Resistance to ascites syndrome, homoeothermic competence and levels of Hsp70 in the heart and lung of broilers. Revistabrasileira de zootecnia-brazilian. J Anim Sci.

[45] Zulkifli I, Omar AR, Sazili AQ, Rajion MA (2003). Crating and heat stress influence blood parameters and heat shock protein 70 expression in broiler chickens showing short or long tonic immobility reactions. Anim Welf.

[46] Taylor P, Zulkifli I, Norma MTC, Israf DA, Omar AR (2010). The effect of early-age food restriction on heat shock protein 70 response in heat-stressed female broiler chickens. Br Poult Sci.

[47] Wang S, Edens FW (1993). Stress-induced heat-shock protein synthesis in peripheral leukocytes of turkeys, meleagris gallopavo. Comp Biochem Physiol.

[48] Lewis S, Handy RD, Cordi UB, Billinghurst Z, Depledge MH (2000). Stress proteins (HSP’s): methods of detection and their use as an environmental biomarker. Ecotoxicology.

[49] Mosser DD, Caron AW, Bourget L, Meriin AB, Sherman MY, Morimoto RI (2000). The chaperone function of hsp70 is required for protection against stress-induced apoptosis. Mol Cell Biol.

[50] Stankiewicz AR, Lachapelle G, Foo CP, Radicioni SM, Mosser DD (2005). Hsp70 inhibits heat-induced apoptosis upstream of mitochondria by preventing Bax translocation. J Biol Chem.

[51] Lee JM, Kim KR, Im H, Kim YH (2015). Zinc preconditioning protects against neuronal apoptosis through the mitogen-activated protein kinase-mediated induction of heat shock protein 70. Biochem Biophys Res Commun.

[52] Azad MAK, Kikusato M, Sudo S, Amo T, Toyomizu M (2010). Time course of ROS production in skeletal muscle mitochondria from chronic heat-exposed broiler chicken. Comp Biochem Physiol A Mol Integr Physiol.

[53] Gu ZT, Li L, Wu F, Zhao P, Yang H, Liu YS (2015). Heat stress induced apoptosis is triggered by transcription-independent p53, Ca(2+) dyshomeostasis and the subsequent Bax mitochondrial translocation. Sci Rep.

[54] Guerin P, El-Mouatassim S, Menezo Y (2001). Oxidative stress and protection against reactive oxygen species in the pre-implantation embryo and its surroundings. Hum Reprod.

[55] Fu Y, He CJ, Ji PY, Zhuo ZY, Tian XZ, Wang F (2014). Effects of melatonin on the proliferation and apoptosis of sheep granulosa cells under thermal stress. Int J Mol Sci.

[56] Cabello CM, Bair WB, Lamore SD, Ley S, Bause AS, Azimian S (2009). The cinnamon-derived Michael acceptor Cinnamic aldehyde impairs melanoma cell proliferation, invasiveness, and tumor growth. Free Rad Biol Med.

[57] Wu KC, McDonald PR, Liu JJ, Chaguturu R, Klaassen CD (2012). Implementation of a high-throughput screen for identifying small molecules to activate the Keap1-Nrf2-ARE pathway. PLoS One.

[58] Alemu TW, Hari OP, Dessie SW, Samuel G, Christiane N, Ernst T (2017). Oxidative and endoplasmic reticulum stress defense mechanisms of bovine granulosa cells exposed to heat stress. Theriogenology.

[59] Alcendor RR, Gao S, Zhai P, Zablocki D, Holle E, Yu X (2007). Sirt1 regulates aging and resistance to oxidative stress in the heart. Circ Res.

[60] Fernando WGD, Ramarathnam R, Krishnamoorthy AS, Savchuk S (2005). Identification and use of bacterial organic volatiles in biological control of Sclerotiniasclerotiorum. Soil Biol Biochem.

[61] Vesco DAP, Gasparino E (2013). Production of reactive oxygen species, gene expression, and enzymatic activity in quail subjected to acute heat stress. J Anim Sci.

[62] Wolfenson D, Lew BJ, Thatcher WW, Graber Y, Meidan R (1997). Seasonal and acute heat stress effects on steroid production by dominant follicles in cows. Anim Reprod Sci.

[63] Mendelson CR, Jiang B, Shelton JM, Richardson JA, Hinshelwood MM (2005). Transcriptional regulation of aromatase in placenta and ovary. J Steroid Biochem Mol Biol.

[64] Mosa A, Neunzig J, Gerber A, Zapp J, Hannemann F, Pilak P (2015). 2β-and16β-hydroxylase activity of CYP11A1 and direct stimulatory effect of estrogens on pregnenolone formation. J Steroid Biochem Mol Biol.

[65] Zhang JY, Wu Y, Zhao S, Liu ZX, Zeng SM, Zhang GX (2015). Lysosomes are involved in induction of steroidogenic acute regulatory protein (StAR) gene expression and progesterone synthesis through low-density lipoprotein in cultured bovine granulosa cells. Theriogenology.

[66] Rekawiecki R, Nowik M, Kotwica J (2005). Stimulatory effect of LH, PGE2 and progesterone on StAR protein, cytochrome P450 cholesterol side chain cleavage and 3beta hydroxysteroid dehydrogenase gene expression in bovine luteal cells. Prostaglandins Other Lipid Mediat.

[67] Sirotkin AV, Bauer M (2011). Heat shock proteins in porcine ovary: synthesis, accumulation and regulation by stress and hormones. Cell Stress Chaperones.

[68] Shimizu T, Ohshima I, Ozawa M, Takahashi S, Tajima A, Shiota M (2005). Heat stress diminishes gonadotropin receptor expression and enhances susceptibility to apoptosis of rat granulosa cells. Reproduction.

[69] Saibil H (2013). Chaperone machines for protein folding, unfolding and disaggregation. Nature reviews Mol Cell Biol.

[70] Livak KJ, Schmittgen TDF (2001). Analysis of relative gene expression data using real-time quantitative PCR and the 2(−Delta Delta C(T)) method. Methods (San Diego Calif).

