# Supplementary material for: Evaluation of heat stress effects on cellular and transcriptional adaptation of bovine granulosa cells
Source: J Anim Sci Biotechnol. 2020 Feb 18;11:25. doi: 10.1186/s40104-019-0408-8 (PMC7027041; doi:10.1186/s40104-019-0408-8)
Supplement: Supplementary file 5 — Additional file 5: Table of gene primers for RT-qPCR. [file 40104_2019_408_MOESM5_ESM.docx]

| Gene | Accession No. | Forward 5′→3′ | Reverse 5′→3′ |
| --- | --- | --- | --- |
| *STAR* | NM_174189.3 | CCCATGGAGAGGCTTTATGA | TGATGACCGTGTCTTTTCCA |
| *BAX* | NM_173894.1 | AGACACCTGAGCTGACCTTG | GTCCCGAAGTAGGAGAGGAG |
| *CASP-3* | NM_001077840.1 | CTGGACTGTGGCATTGAGAC | GCAAAGGGACTGGAGAACC |
| *CYP11A1* | NM_176644.2 | CTGGCATCTCCACAAAGACC | GTTCTCGATGTGGCGAAAGT |
| *CAT* | NM_001035386.2 | GTTCGCTTCTCCACTGTTGC | AGGTGCGTTTGAGGGTTTCT |
| *BCL2L1* | NM_001077486.2 | CGATGAAGGGGGTATGTGGC | TTCAAACTCATCGCCTGCCT |
| *PCNA* | NM_001034494.1 | GCGTTCATAGTCGTGTTCCG | TTCAAGATGGAGCCCTGGAC |
| *SOD2* | NM_201527.2 | TCAATAAGGAGCAGGGACGC | AAGCCGTGTATCGTGCAGTT |
| *FOXO3* | NM_001206083.1 | TCCAGACAAACGGCTCACTC | AGTGTCTGGTTGCCGTAGTG |
| *KEAP1* | NM_001101142.1 | AGATTGACCAGCAGAACTGTACCT | GGCTCACGAGTTCCCACTCTA |
| *INHBA* | NM_174363.2 | AGAACTCTCCTCCCTGACAGC | ATCCAGCAACTCGCCAACAA |
| *MAPK8IP1* | NM_001102508.1 | GCCCAATTTCAGGCTCACCC | CTGATGCCACACTCATCGGT |
| *CDK2* | NM_001014934.1 | TCTTTGCTGAGATGGTGACCC | AGGGTCGTAGTGCAGCATTT |
| *HMOX1* | NM_001014912.1 | GGGGCCATGAAAACTGTTCG | TGGTGGAGATGTCTCAGGCT |
| *HSPA13* | NM_001038505.2 | TGACAGAAAGGGACCTCCGA | CAGTCTTTTCCAGGTGGCCT |
| *GAPDH* | NM_001034034.2 | GGTGCTGAGTATGTGGTGGA | GGCATTGCTGACAATCTTGA |

**Primers used for RT-qPCR validation of RNA-Seq data**
