# Supplementary material for: Evaluation of heat stress effects on cellular and transcriptional adaptation of bovine granulosa cells
Source: J Anim Sci Biotechnol. 2020 Feb 18;11:25. doi: 10.1186/s40104-019-0408-8 (PMC7027041; doi:10.1186/s40104-019-0408-8)

**Figure legends**

**Fig. 1. Functional annotation cluster and gene ontology analysis along three groups:** Summary of GO terms for biological process (BP), molecular function (MF), and cellular component (CC) ontologies for DEGs products in response to heat stress under the comparison of Control vs 39°C (a,b,c), Control vs 40°C (d,e,f) and Control vs 41°C (g,h,i).

**Fig. 2. Validation of RNA-Seq results by RT-qPCR :** Comparison of RT-qPCR findings to RNA-Seq results. Validation of differentially expressed genes by RT-qPCR. Relative quantification of 15 representative genes was performed. qRT-PCR values were determined from the ^ΔΔ^ Ct for the target genes.

**Fig. 1.**


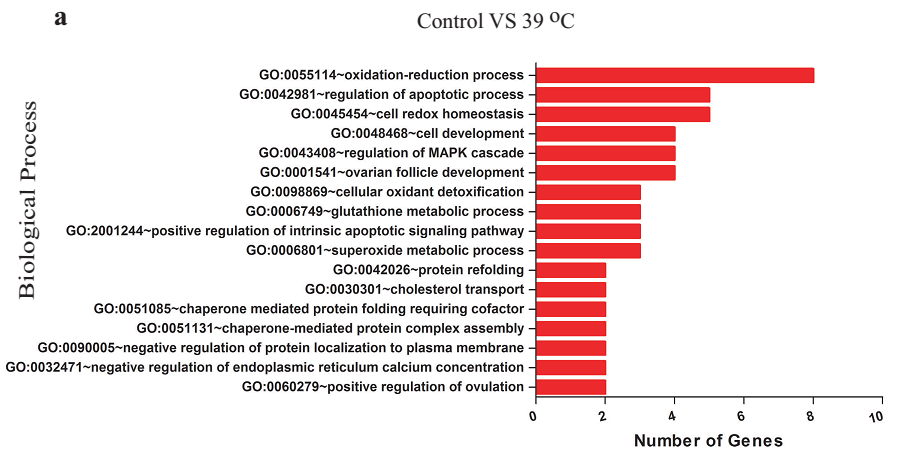


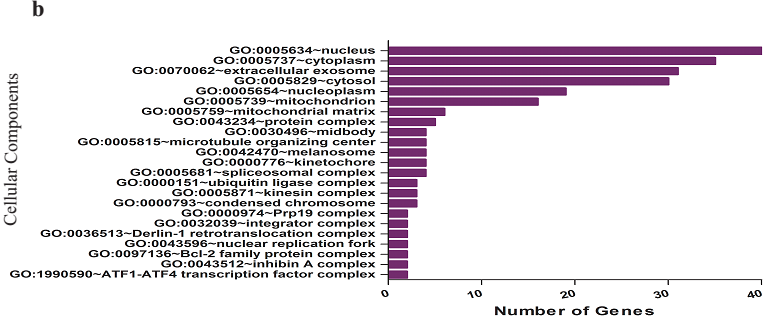


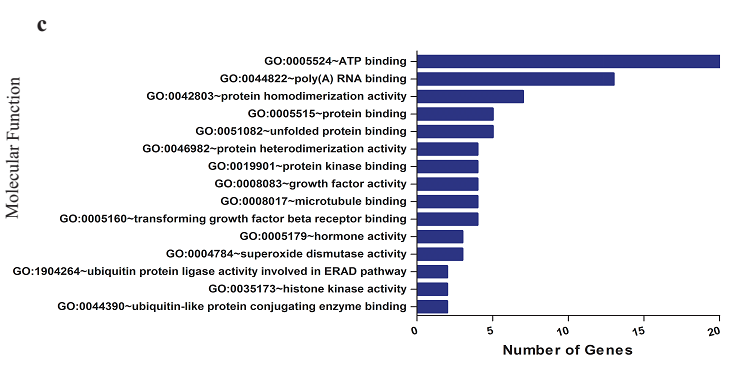


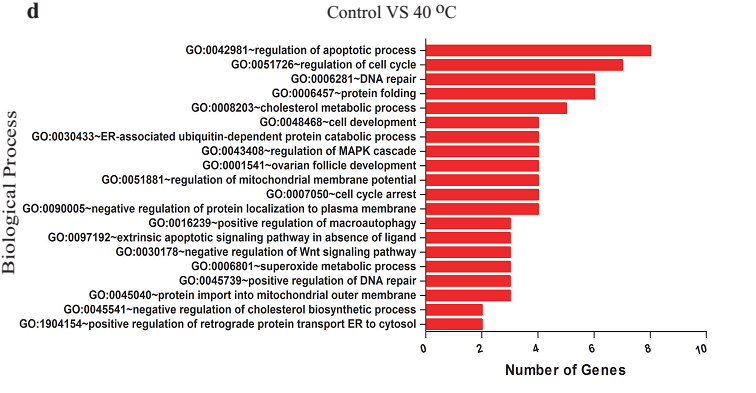


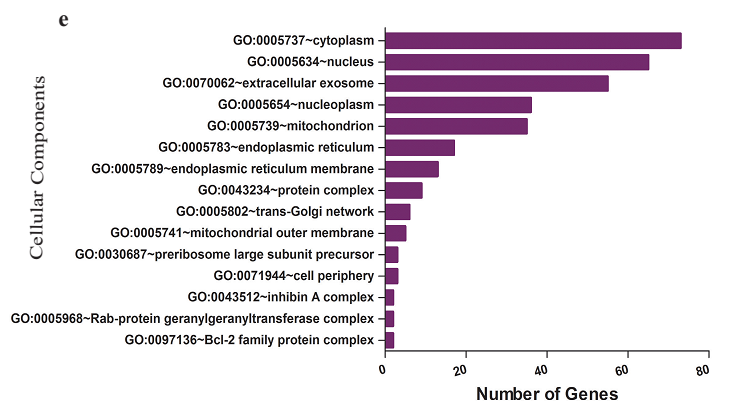


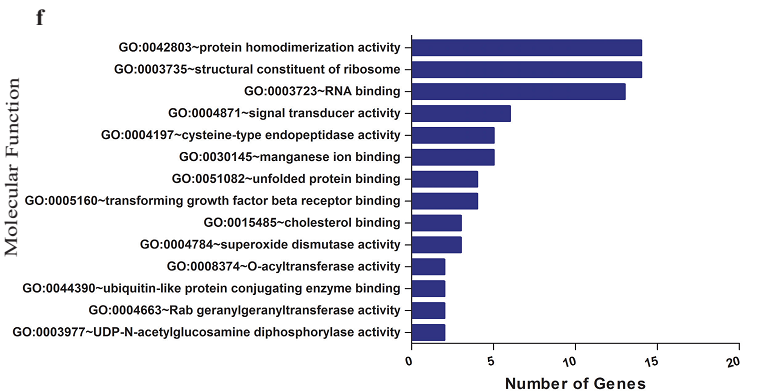


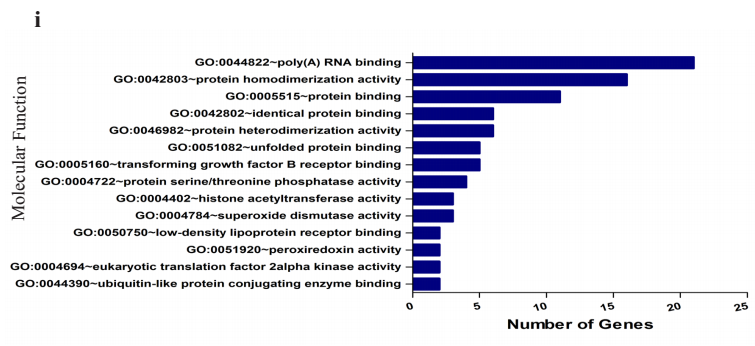

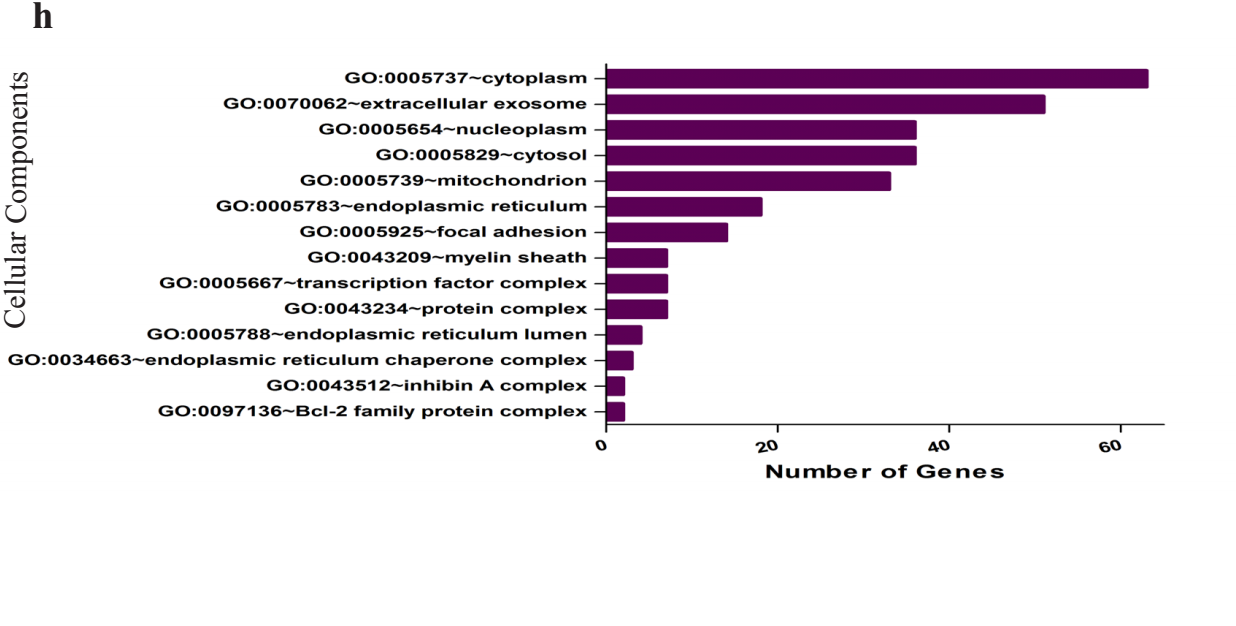

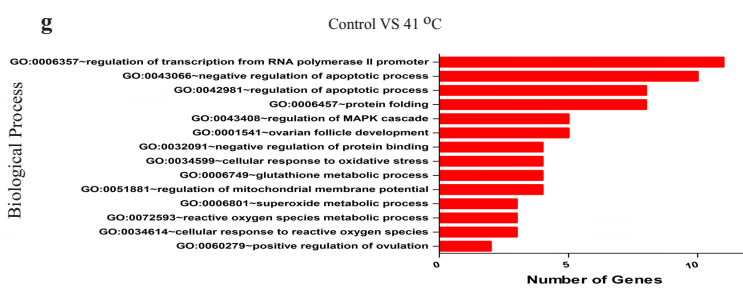


**Fig. 2.**


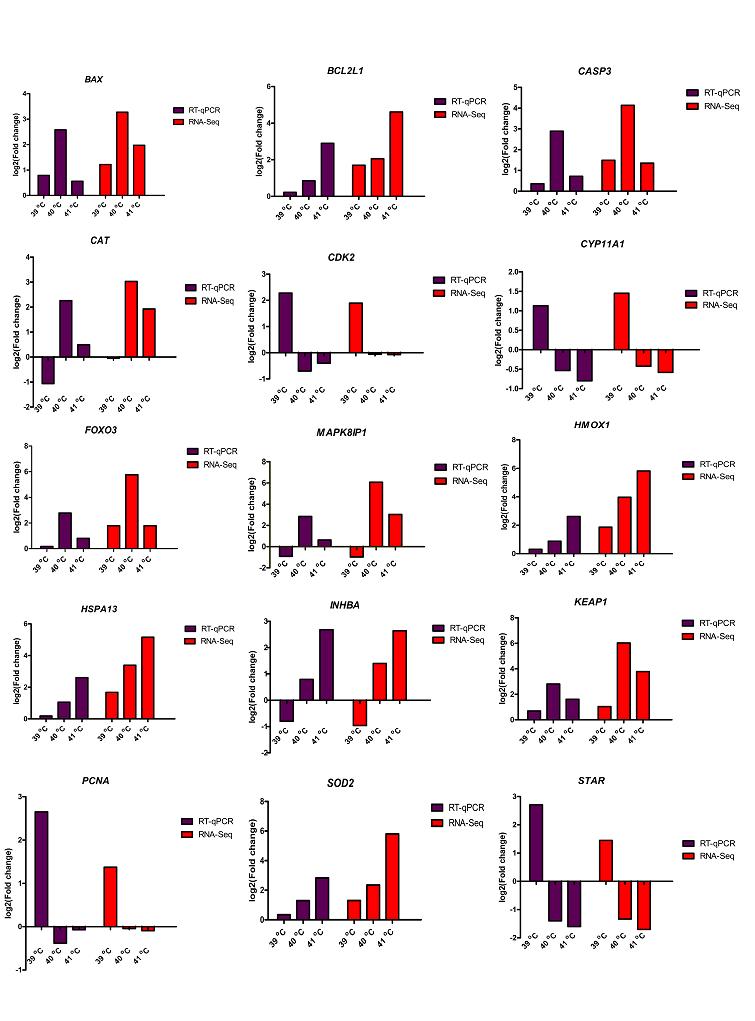

Supplement: Supplementary file 6 — Additional file 6: Figures. [file 40104_2019_408_MOESM6_ESM.docx]
