# Supplementary figures and images for: Evaluation of heat stress effects on cellular and transcriptional adaptation of bovine granulosa cells
Source: J Anim Sci Biotechnol. 2020 Feb 18;11:25. doi: 10.1186/s40104-019-0408-8 (PMC7027041; doi:10.1186/s40104-019-0408-8)

**
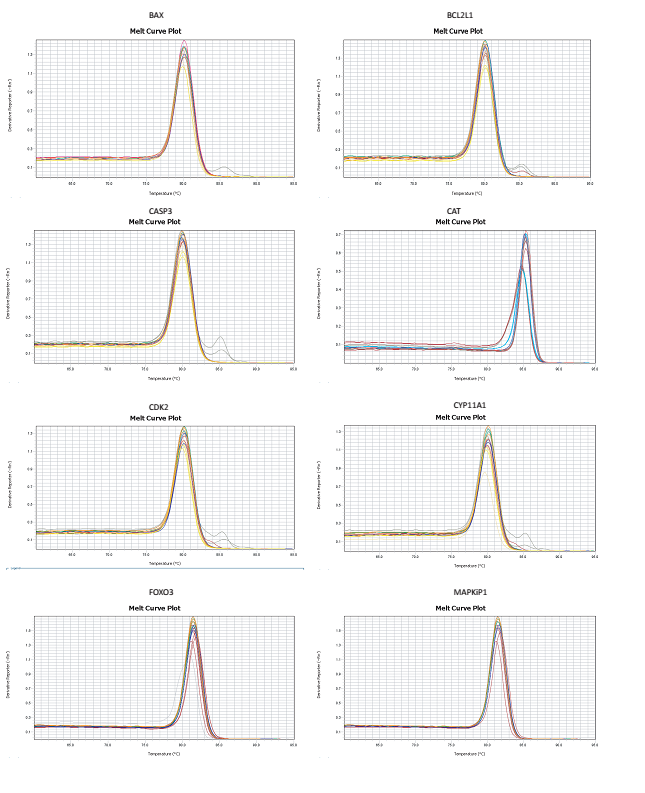
Melting Curve for each primer against GADPH reference gene**


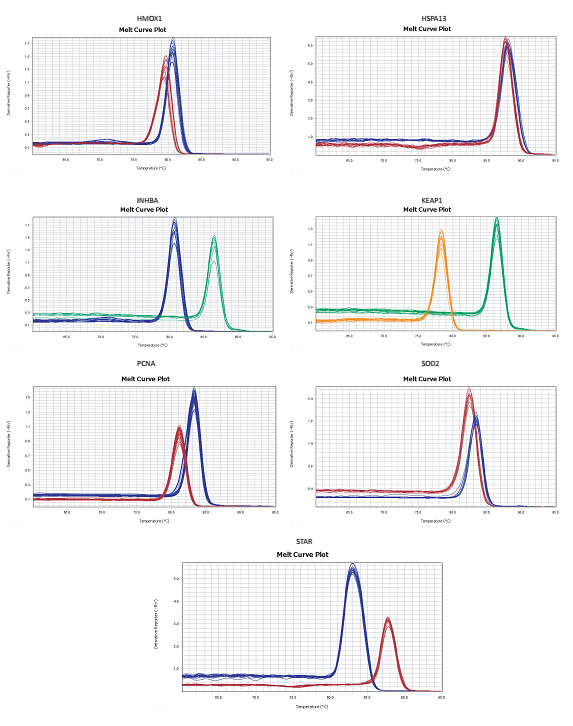

Supplement: Supplementary file 7 — Additional file 7: Covering letter. [file 40104_2019_408_MOESM7_ESM.docx]
